# tRNA Methyltransferase Homolog Gene *TRMT10A* Mutation in Young Onset Diabetes and Primary Microcephaly in Humans

**DOI:** 10.1371/journal.pgen.1003888

**Published:** 2013-10-31

**Authors:** Mariana Igoillo-Esteve, Anne Genin, Nelle Lambert, Julie Désir, Isabelle Pirson, Baroj Abdulkarim, Nicolas Simonis, Anais Drielsma, Lorella Marselli, Piero Marchetti, Pierre Vanderhaeghen, Décio L. Eizirik, Wim Wuyts, Cécile Julier, Ali J. Chakera, Sian Ellard, Andrew T. Hattersley, Marc Abramowicz, Miriam Cnop

**Affiliations:** 1 Laboratory of Experimental Medicine, Université Libre de Bruxelles, Brussels, Belgium; 2 IRIBHM, Université Libre de Bruxelles, Brussels, Belgium; 3 Genetics Department, Hôpital Erasme, Université Libre de Bruxelles, Brussels, Belgium; 4 Laboratoire de Bioinformatique des Génomes et des Réseaux (BiGRe), Université Libre de Bruxelles, Brussels, Belgium; 5 Department of Clinical and Experimental Medicine, Islet Laboratory, Cisanello University Hospital, Pisa, Italy; 6 Department of Medical Genetics, University of Antwerp and Antwerp University Hospital, Edegem, Belgium; 7 Inserm UMR-S958, Faculté de Médecine Paris Diderot, Paris, France; 8 University Paris 7 Denis-Diderot, Paris, France; 9 University of Exeter Medical School, University of Exeter, Exeter, United Kingdom; 10 Division of Endocrinology, Erasmus Hospital, Brussels, Belgium; Max Planck Institute for Molecular Genetics, Germany

## Abstract

We describe a new syndrome of young onset diabetes, short stature and microcephaly with intellectual disability in a large consanguineous family with three affected children. Linkage analysis and whole exome sequencing were used to identify the causal nonsense mutation, which changed an arginine codon into a stop at position 127 of the tRNA methyltransferase homolog gene *TRMT10A* (also called *RG9MTD2*). TRMT10A mRNA and protein were absent in lymphoblasts from the affected siblings. TRMT10A is ubiquitously expressed but enriched in brain and pancreatic islets, consistent with the tissues affected in this syndrome. In situ hybridization studies showed that TRMT10A is expressed in human embryonic and fetal brain. *TRMT10A* is the mammalian ortholog of *S. cerevisiae TRM10*, previously shown to catalyze the methylation of guanine 9 (m^1^G_9_) in several tRNAs. Consistent with this putative function, in silico topology prediction indicated that TRMT10A has predominant nuclear localization, which we experimentally confirmed by immunofluorescence and confocal microscopy. TRMT10A localizes to the nucleolus of β- and non-β-cells, where tRNA modifications occur. TRMT10A silencing induces rat and human β-cell apoptosis. Taken together, we propose that TRMT10A deficiency negatively affects β-cell mass and the pool of neurons in the developing brain. This is the first study describing the impact of TRMT10A deficiency in mammals, highlighting a role in the pathogenesis of microcephaly and early onset diabetes. In light of the recent report that the type 2 diabetes candidate gene *CDKAL1* is a tRNA methylthiotransferase, the findings in this family suggest broader relevance of tRNA methyltransferases in the pathogenesis of type 2 diabetes.

## Author Summary

The inherited predisposition to type 2 diabetes is attributed to common variants in over 60 loci. Among these risk variants is *CDKAL1*, which has recently been shown to be a tRNA modifying enzyme (methylthiotransferase). Genetic variants of different severity can generate a spectrum of monogenic and polygenic forms of diabetes. Here we describe a new syndrome of young onset diabetes, short stature and microcephaly (small brain size) with intellectual disability in a large consanguineous family. By linkage analysis and whole exome sequencing we identified a nonsense mutation in *TRMT10A*, a gene that has hitherto not been studied in mammals. The yeast homolog TRM10 has been shown to be a tRNA modifying enzyme with methyltransferase activity. We demonstrate that TRMT10A mRNA and protein are absent in cells from the affected siblings. TRMT10A localizes to the nucleolus, where tRNA modifications occur. TRMT10A silencing induces cell death in insulin-producing pancreatic β-cells, suggesting that TRMT10A deficiency may reduce β-cell mass and the pool of neurons in the brain. This is the first study describing the impact of TRMT10A deficiency in man. Our findings may have broader relevance for the understanding of the pathogenesis of type 2 diabetes and microcephaly.

## Introduction

Type 2 diabetes (T2D) is a heterogeneous polygenic disease with dramatically increasing worldwide incidence as a consequence of the obesity epidemic [1]. Environmental factors (energy dense diets rich in saturated fat and sedentary lifestyle [2], [3]) and genetic predisposition contribute to its pathogenesis. T2D develops when β-cells fail to compensate for peripheral insulin resistance by increasing insulin secretion [4], [5] as a consequence of β-cell dysfunction and reduced β-cell mass. Genome-wide association studies have identified a number of loci where genetic polymorphisms associate with T2D [6]. Inherited mutations in genes at some of these loci have been shown to cause monogenic forms of diabetes, indicating that genetic variants of different severity can generate a spectrum of monogenic and polygenic forms of diabetes [7]. An example of a T2D risk gene is *CDK5* regulatory associated protein 1-like 1 (*CDKAL1*). Polymorphisms in this gene have been associated with T2D across ethnic populations [8]. *CDKAL1* encodes a transfer RNA (tRNA) methylthiotransferase that catalyzes the methylthiolation of tRNA^Lys^(UUU) [9]. Cdkal1-deficient β-cells have impaired glucose-induced insulin secretion, and Cdkal1 knockout mice develop glucose intolerance due to aberrant insulin synthesis [9].

tRNAs undergo modifications of their bases or sugar moieties that are crucial for proper cellular function. Mammalian cells have an average of 13–14 modifications per tRNA [10]–[12], methylation being the most common one [12]. Chemical modifications of nucleotides surrounding anticodons of tRNAs are important to preserve translational efficiency and fidelity [13], modifications in the main body of the tRNA affect its folding and stability, and other modifications at various positions influence tRNA identity [14], [15].

Here we identified a nonsense mutation in *TRMT10A* (also called *RG9MTD2*) in a new syndrome of young onset diabetes and microcephaly. The *TRMT10A* yeast ortholog *YOL093w* codes for the protein TRM10 that has tRNA methyltransferase activity. TRM10 specifically methylates tRNA-Arg, -Asn, -Gln, -Thr, -Trp, -Met and -Lys at position 9 (m^1^G_9_), using S-adenosylmethionine (SAM) as methyl donor [16]. TRM10 was shown to be the major if not the only m^1^G_9_ methyltransferase in yeast, but its knockout did not alter cell survival or growth [16]. Mutational analysis in yeast revealed potential interactions between TRM10, TRM8/TRM82, and TRM1 [17]. These latter proteins have tRNA methyltransferase activity towards m^7^G_46_ and m^2^_2_G_26_, respectively [12]. The concomitant deletion of *TRM10* with *TRM8*, *TRM82* or *TRM1* induced growth arrest in *S. cerevisiae* exposed to high temperature, suggesting enhanced tRNA instability [17].

Here we describe the affected siblings and the identification of the *TRMT10A* mutation. We followed this up with studies of TRMT10A expression in tissues and subcellular localization, and interrogated the functional consequences of TRMT10A deficiency.

## Results

### Description of the patients

**Figure 1:**
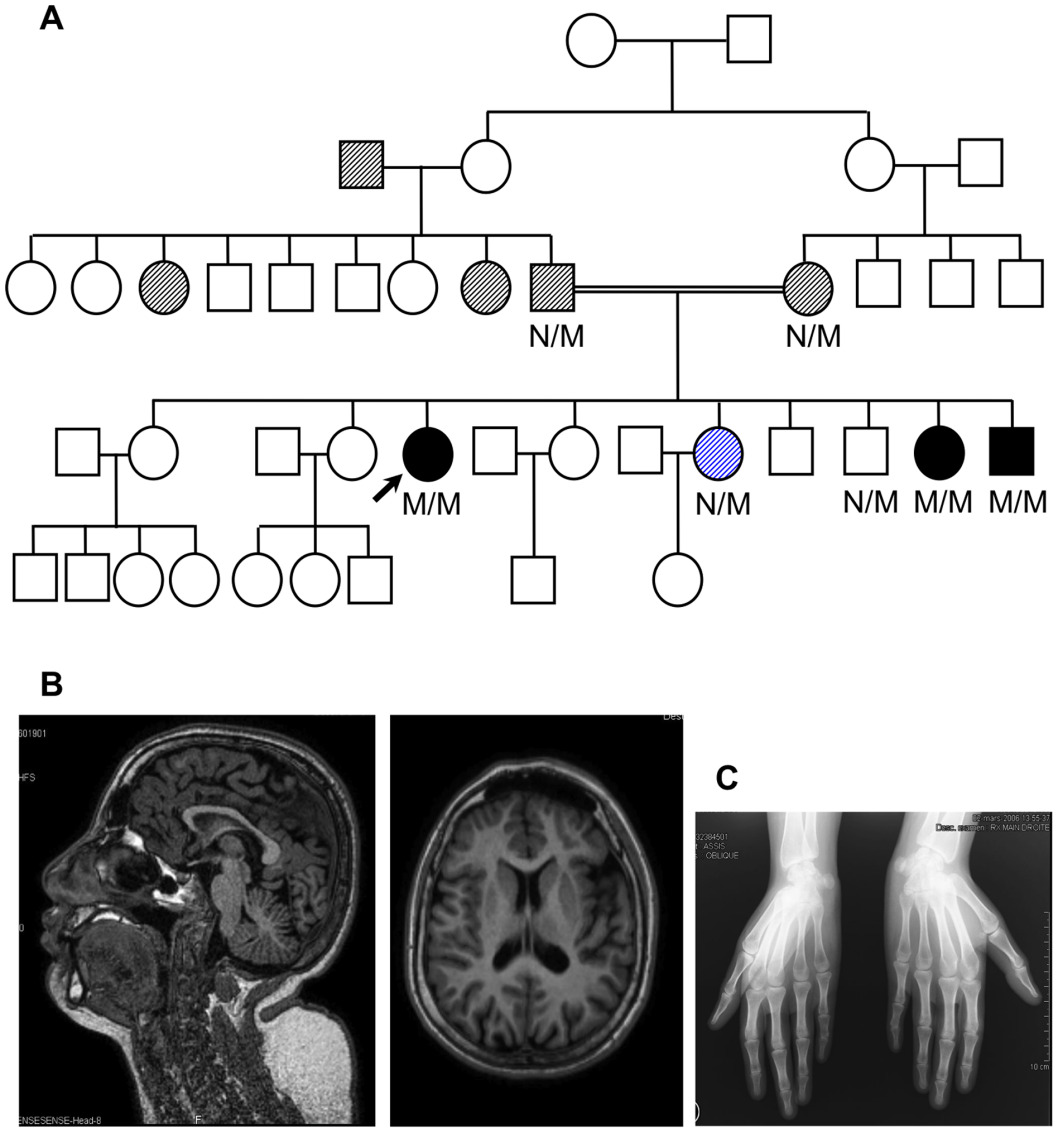
Diabetes and microcephaly in a large consanguineous family. (A) Three siblings presented with young onset diabetes and microcephaly with intellectual disability (black symbols); arrow shows the proband. Hatched symbols represent relatives with adult onset diabetes and the blue hatched symbol gestational diabetes. The double line indicates consanguinity. M/M denotes two mutant alleles, N/M one normal and one mutant allele. (B) A head MRI in the proband at age 26 years showed a small brain with normal architecture and normal gyration. (C) The radiograph of the hands showed normally shaped bones and absence of epiphyseal dysplasia.

The proband was born to consanguineous parents of Moroccan origin, her paternal and maternal grandmothers being sisters (Figure 1). Head circumference, weight and length at birth are unknown. At age 26 years she had short stature (143 cm), microcephaly (adult head circumference 49 cm, -5SD) and intellectual disability, with a history of petit mal epilepsy in adolescence. Magnetic resonance imaging of the head showed a small brain with no malformation or other abnormality (Figure 1). She had developed diabetes at the age of 22 years. At diagnosis her body mass index (BMI) was 26.9 kg/m^2^; plasma glucose was 176 mg/dl and HbA1c 11.3%. Other features were a short neck, wide nose, low hairline, buffalo hump, retraction of the right 5^th^ toe, scoliosis, and joint laxity. She also had osteoporosis, with dual-energy X-ray absorptiometry T-scores of -2.7 and -3.5 at the lumbar spine and femoral neck, respectively. A skeletal survey revealed no epiphyseal dysplasia or other bone abnormality (e.g. normal X-ray of the hands, Figure 1). Her sister had short stature (154 cm), microcephaly (adult head circumference 51 cm, -3SD) and intellectual disability (IQ 69). She developed diabetes at the age of 19 years, presenting with a fasting glucose of 365 mg/dl and HbA1c 13.2%. Her BMI was 21.7 kg/m^2^. A younger brother had short stature (141 cm at age 14 years and final height of 157 cm at 21 years), microcephaly (head circumference 51 cm, -3SD) and mental retardation (IQ 52). His head circumference at birth was reportedly normal (36 cm). He was diagnosed with diabetes at 14 years of age, with a plasma glucose of 251 mg/dl and HbA1c 11.1%. His BMI was 20.6 kg/m^2^. None of the patients had ketoacidosis and all three were treated with insulin at diagnosis. They were negative for anti-insulin, anti-GAD65, anti-IA2 and islet cell autoantibodies and had a HLA genotype that did not confer risk for type 1 diabetes. Endogenous insulin secretion persisted, shown by C-peptide measurements for up to 20 years of follow-up. The insulin requirements were moderate with an average insulin dose of 0.4–1.2 U/kg/day; glycemic control ranged from good to insufficient (HbA1c 6.5–8.5%). After 18 years of diabetes, the proband's ophthalmologic examination revealed bilateral diabetic retinopathy and cortical cataract. The parents and non-affected siblings had normal size (parents 166 and 157 cm, siblings 160, 175, 183 and 159 cm) and head circumference (both parents 58 cm, P97). The parents developed diabetes at age 58 years (BMI 30.9 and 31.6 kg/m^2^, plasma glucose 124 and 169 mg/dl and HbA1c 8.3 and 7.6% in the mother and father, respectively) and were treated with metformin and a sulphonylurea. One grandfather and two aunts had adult onset diabetes (Figure 1). One sister had gestational diabetes at the age of 22 years; her fasting plasma glucose was normal (90 mg/dl) at age 30 (Figure 1).

### Identification of the mutation

The GeneChip SNP array analysis identified only one large (>3 cM) homozygous genomic region that was common to the three affected siblings. It was located on chromosome 4q22-23 and spanned 12.4 Mb between heterozygous SNPs rs4128340 and rs10516462. In this segment, we genotyped 15 microsatellite markers, which confirmed homozygosity and biparental inheritance of a haplotype shared by both parents (Figure S1). The multipoint LOD score was 3.0. Microsatellite analysis in the unaffected sister with a history of gestational diabetes (Figure 1) showed inheritance of the non-mutated maternal haplotype and of the mutated paternal haplotype. In an additional unaffected brother with normal fasting plasma glucose (84 mg/dl) and HbA1c (5.1%) at age 23 years, we observed a critical meiotic recombination event, resulting in homozygosity for all disease-associated markers except those distal to microsatellite D4S1628. This recombinant chromosome reduced the critical linkage region to a 3.1 Mb segment at 4q23.

We initially sequenced the following genes located in the 3.1 Mb segment and considered as candidates: H2AFZ (H2A histone family, member Z), LAMTOR3 (late endosomal/lysosomal adaptor, MAPK and MTOR activator 3), DDIT4L (DNA-damage-inducible transcript 4-like), RAP1GDS1 (RAP1, GTP-GDP dissociation stimulator 1) and METAP1 (methionyl aminopeptidase 1), but no mutation was identified.

**Figure 2:**
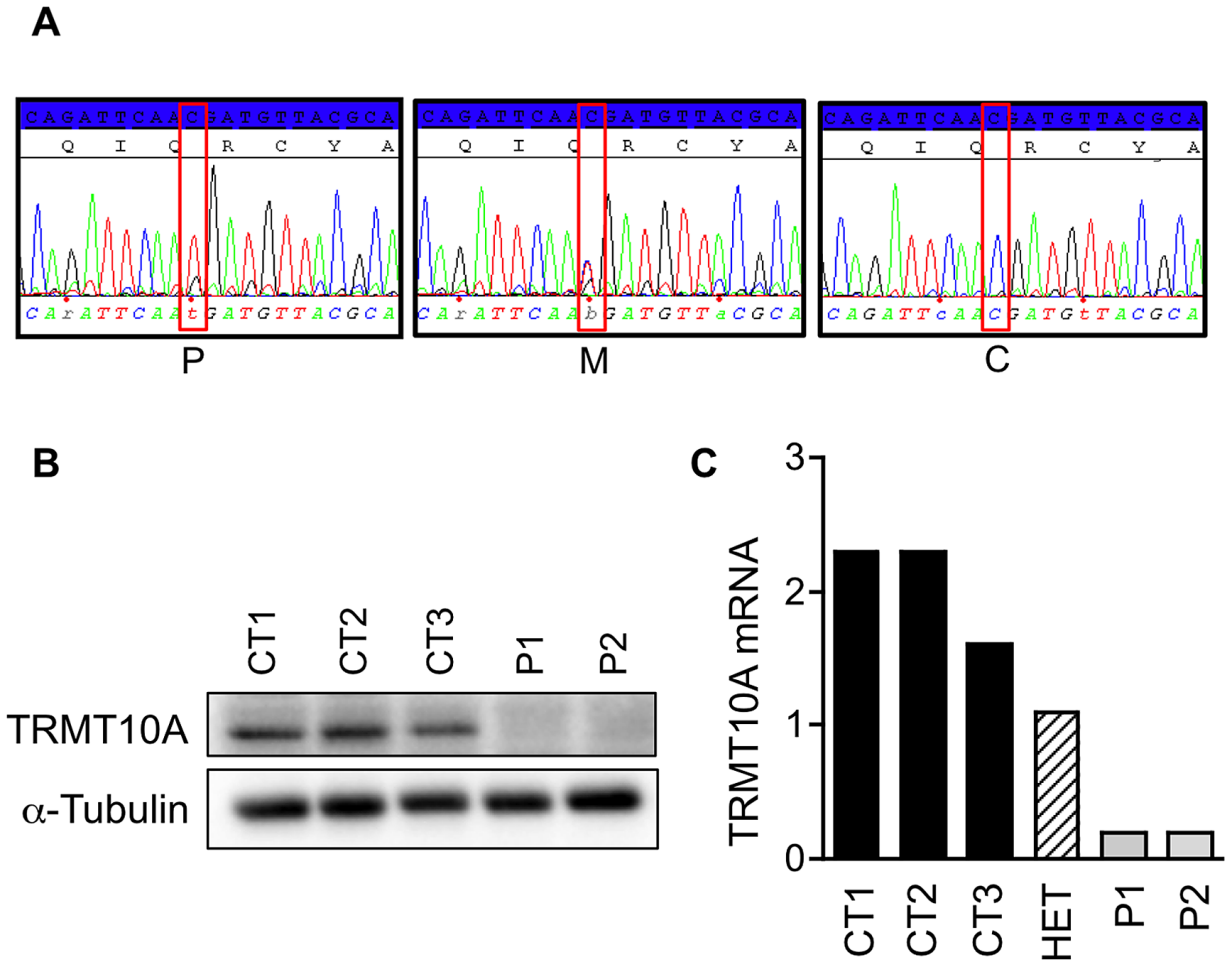
Patients are homozygous for a nonsense mutation in TRMT10A and lose TRMT10A expression. (A) Sanger sequencing at the level of the mutation identified by whole exome sequencing in proband. The mutation c.379 G>A; p.Arg127Stop changes a CGA codon (Arginine) into a TGA codon (Stop), and is found homozygous in the proband (P), heterozygous in unaffected mother (M) and absent in an unrelated control subject (C). (B) TRMT10A protein and (C) mRNA expression was examined by Western blot and real-time PCR in lymphoblast from three controls (CT1-3), two patients homozygous for the nonsense mutation (P1 and P2) and one heterozygous carrier (HET). α-Tubulin was used as loading control and TRMT10A mRNA expression was normalized to the geometric mean of the reference genes GAPDH, actin and OAZ1 expression.

Exonic sequences-enriched DNA (whole exome) sequencing was performed in one proband and results were analyzed for variants that were not found in: dbSNP135 database, the Thousand Genomes database, the Exome Variant Server, or in-house exome sequencing on 51 individuals. There was only a single candidate mutation in the 3.1 Mb critical linkage segment, a homozygous G to A transition in exon 4 of gene *TRMT10A* (tRNA methyltransferase 10 homolog A (*S. cerevisiae*) at position 379 of the coding DNA sequence, predicted to replace an Arginine residue with a premature termination codon at position 127 of the polypeptide (c.379 G>A; p.Arg127Stop). Sanger sequencing confirmed the mutation (Figure 2A), which was homozygous in the three affected patients and heterozygous in both parents as well as in the unaffected brother with the critical recombination event. A comparison across species shows that Arg127 and the surrounding region are highly conserved (Figure S2). Outside the linkage region, exome analysis in the proband identified biallelic, potentially damaging mutations in the six following genes: BCLAF1; CES1; EVC2; PTPN22; ST13; ZNF626. As none were concordant in the three affected siblings, we rejected them as candidate mutations.

We sequenced the 8 exons and flanking intronic sequences in 20 patients with a similar phenotype of young onset diabetes associated to intellectual disability, microcephaly, epilepsy, developmental delay and/or short stature, five of whom were born to consanguineous parents, but failed to identify another patient with biallelic disease-causing mutations. We furthermore sequenced *TRMT10A* in 26 patients with non-autoimmune diabetes with onset before 25 years and a positive family history of diabetes, in whom no mutation was identified in known MODY-associated genes, but did not identify any mutation in *TRMT10A*.

### The homozygous nonsense mutation Arg127Stop in the TRMT10A gene induces TRMT10A mRNA silencing and TRMT10A protein deficiency

To examine the outcome of the TRMT10A nonsense mutation on TRMT10A protein and mRNA expression, we performed Western blot and real-time PCR on lymphoblasts from two patients, a heterozygous carrier of the mutation, and three healthy controls. TRMT10A protein was absent in lymphoblasts from patients homozygous for the Arg127Stop mutation (Figure 2B). TRMT10A mRNA expression was much reduced in patients, and intermediate in the carrier (Figure 2C). This finding is consistent with nonsense-mediated mRNA decay induced by the premature translation-termination codon (PTC) and/or by PTC-induced transcriptional silencing of the affected gene, a mechanism known to prevent the synthesis of potentially deleterious truncated proteins [18], [19].

### TRMT10A expression is enriched in brain and pancreatic islets

**Figure 3:**
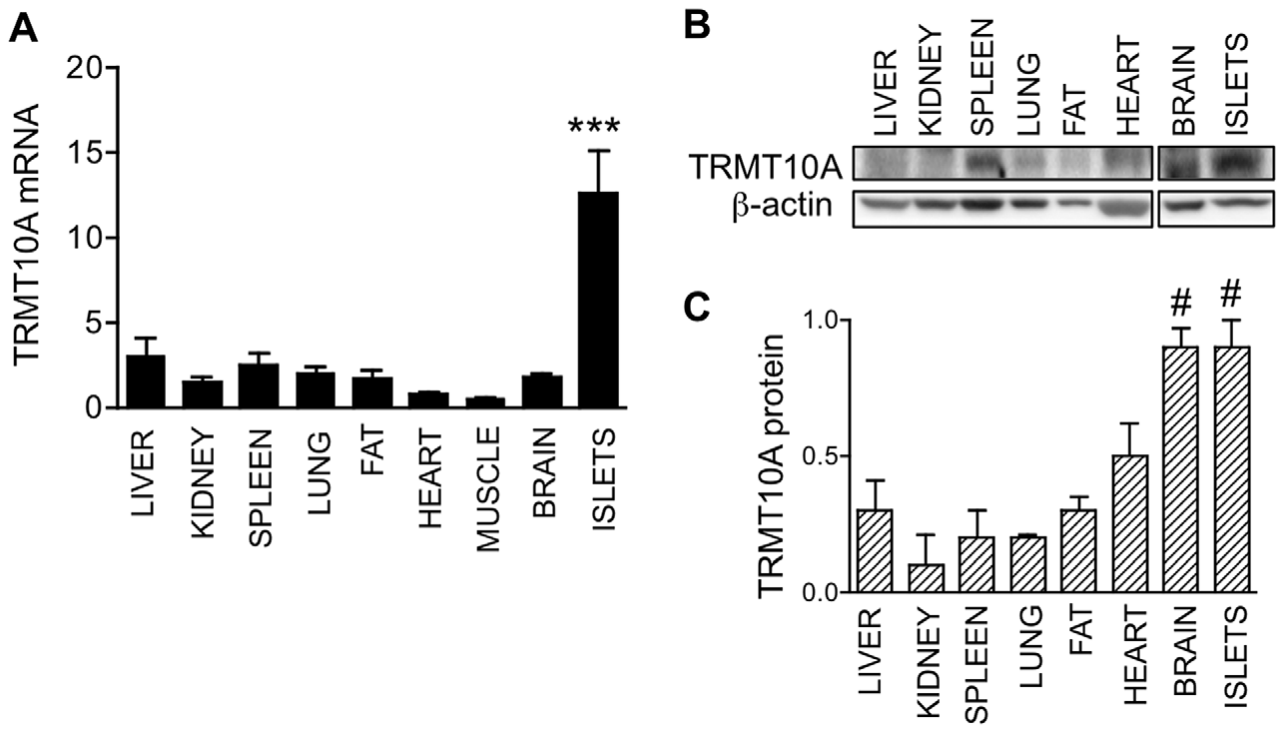
TRMT10A mRNA and protein expression is enriched in brain and pancreatic islets. TRMT10A mRNA (A) and protein (B–C) expression in rat tissues and islets was examined by real-time PCR and Western blot. TRMT10A mRNA expression was normalized to the geometric mean of the reference genes GAPDH, actin, and OAZ1, and protein was normalized to β-actin. Results are means ± SE of n = 3. ***p<0.001 for the comparison islets vs all other tissues, #p<0.05 for islets and brain vs liver, kidney, spleen lung and fat by one-way ANOVA followed by paired *t* test with Bonferroni correction for multiple comparisons.

We next evaluated TRMT10A transcript and protein expression in rat tissues. TRMT10A was ubiquitously expressed with similar mRNA abundance in liver, kidney, spleen, lung, fat, and brain. Heart and muscle showed lesser TRMT10A mRNA expression, while pancreatic islets were enriched in TRMT10A transcripts (Figure 3A). TRMT10A protein was ubiquitously present and 2- to 3-fold more abundant in brain and pancreatic islets compared to other tissues (Figure 3B–C).

### TRMT10A is expressed in human embryonic and fetal brain

**Figure 4:**
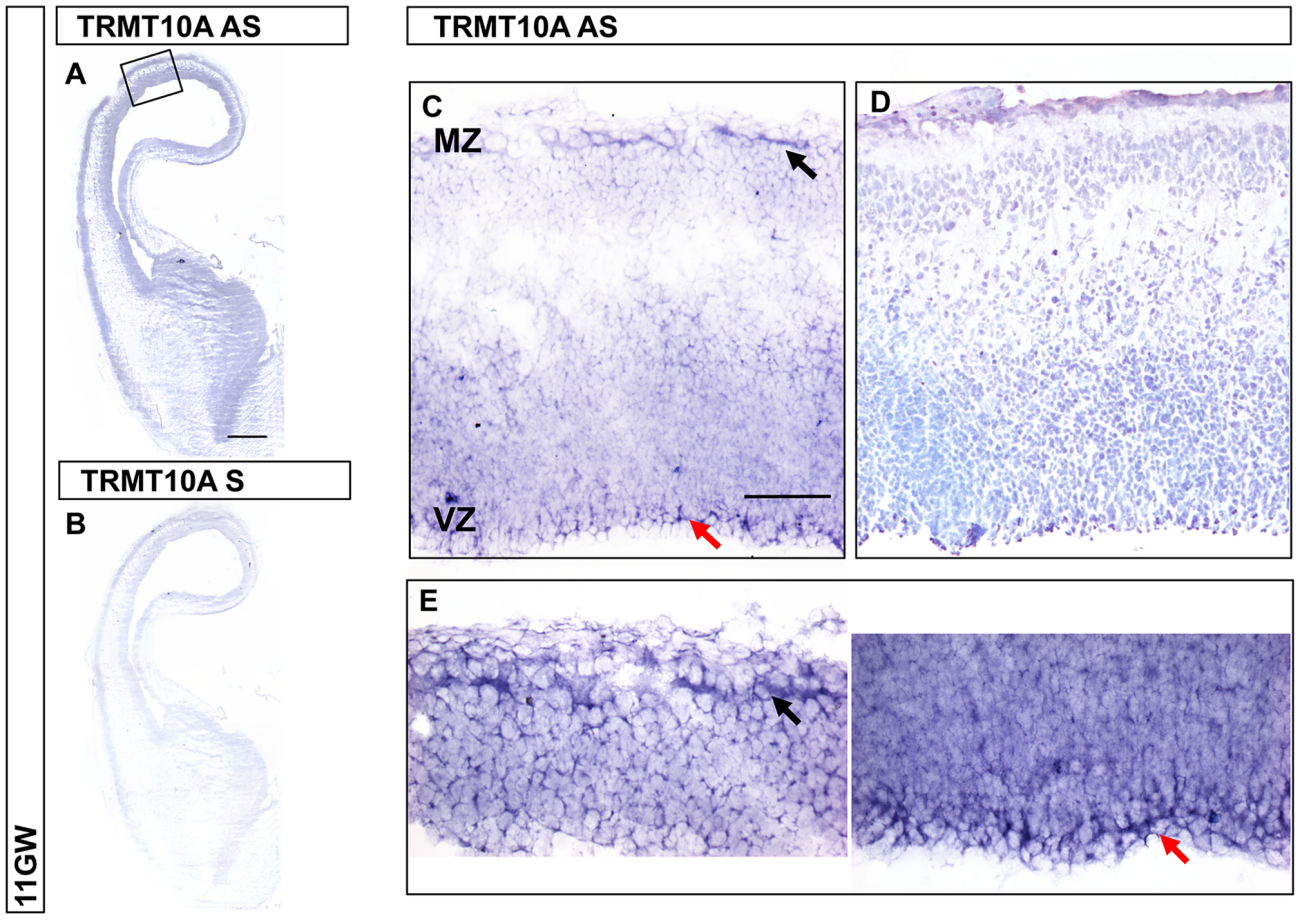
TRMT10A expression profile in fetal telencephalon at 11 GW. (A) TRMT10A antisense (AS) probe and (B) TRMT10A sense (S) probe as a negative control. Scale bar: 1 mm. (C) TRMT10A is expressed throughout the thickness of the dorsal telencephalon at 11 GW, with higher expression in the ventricular zone (VZ, red arrow) and marginal zone (MZ, black arrow). Scale bar: 100 µm. (D) Cresyl Violet staining on adjacent section. (E) Higher resolution of MZ (black arrow) and VZ (red arrow).

*In situ* hybridization studies were performed in human embryonic brain samples at 8, 11, 17 and 19 gestational weeks (GW). *TRMT10A* was expressed throughout the whole thickness of the dorsal telencephalon (presumptive cerebral cortex) at 8 and 11 GW, with higher expression in the ventricular zone and marginal zone (Figure 4). The ventricular zone contains most neural progenitors at early stages of corticogenesis, while the marginal zone is the region where the first post-mitotic neurons migrate. At later stages *TRMT10A* expression was not detected in the dorsal telencephalon but was found in the cerebellar cortex and cerebellar nuclei (Figure S3 and data not shown).

### TRMT10A has predominant nucleolar localization

**Figure 5:**
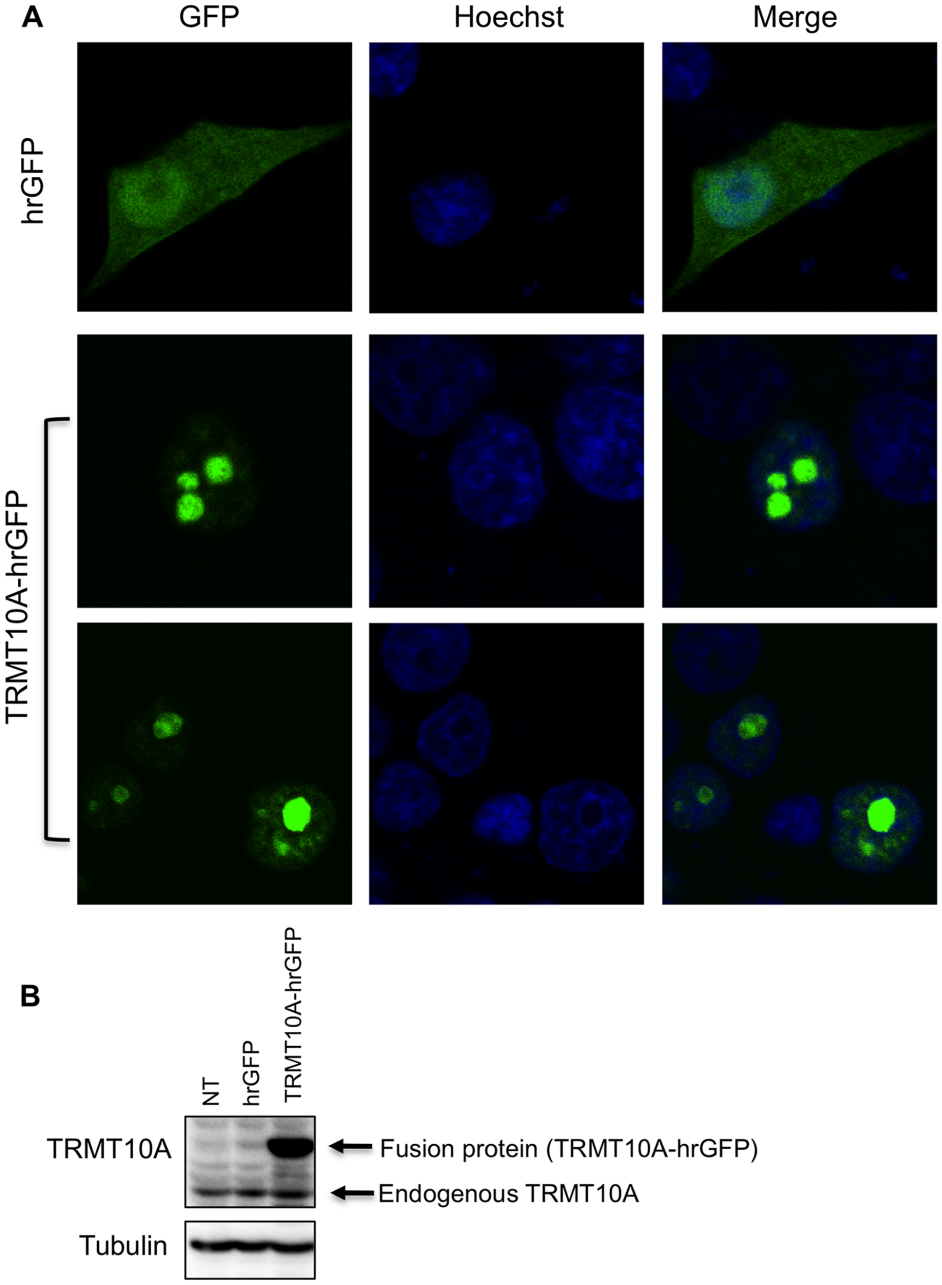
TRMT10A has predominant nuclear localization. INS-1E cells were transfected with a vector encoding hrGFP alone or fused to TRMT10A (TRMT10A-hrGFP). 48 h after transfection RG9MTD2 subcellular localization was examined by confocal microscopy (A). Nuclei were stained with Hoechst 33342. Pictures were taken at 40× magnification, zoom 3×. The presence of the recombinant fusion protein was confirmed by Western blot using an antibody against hTRMT10A (B). NT denotes non-transfected. The figure is representative of three independent experiments.

**Figure 6:**
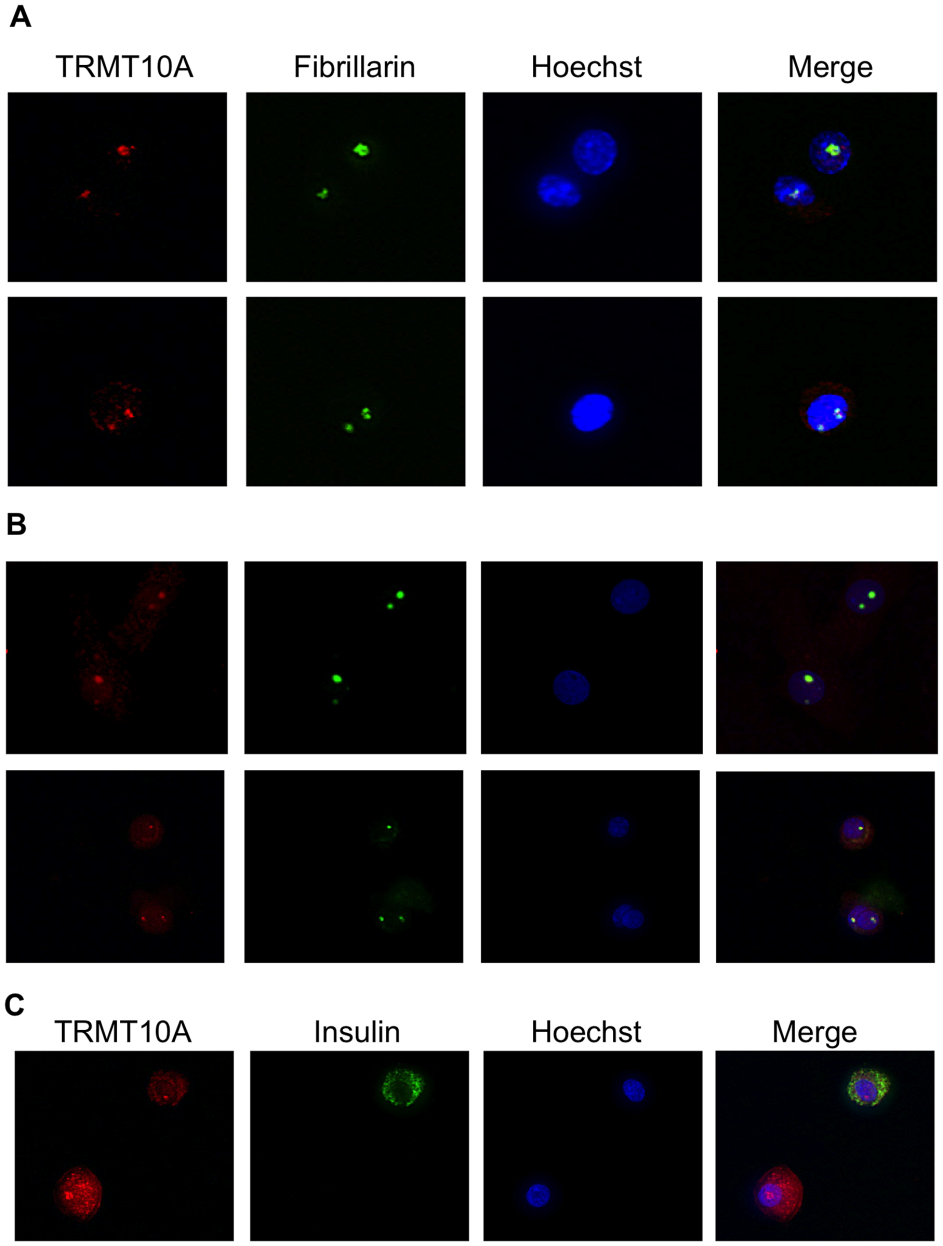
TRMT10A protein expression is enriched in the nucleolus of β- and non-β-cells. Endogenous TRMT10A was detected by immunofluorescence using anti-hTRMT10A antibody in dispersed rat (A) and human islet cells (B–C). Anti-fibrillarin antibody was used to immunolabel the nucleolus. Nuclei were stained with Hoechst 33342. Pictures were taken at 40× magnification and are representative of two independent experiments.

To examine TRMT10A subcellular localization we first performed in silico TRMT10A topology prediction using PSORII and WoLF PSORT [20]. These softwares detected monopartite and bipartite nuclear localization signals in the first 89 amino acids of the protein. This was confirmed with cNLS Mapper [21], [22] suggesting predominant nuclear localization. To experimentally demonstrate the TRMT10A subcellular localization we took two approaches: 1) Expression of a fluorescent recombinant fusion protein, human TRMT10A (hTRMT10A)-humanized Renilla green fluorescent protein (hrGFP); 2) Detection of endogenous TRMT10A by immunofluorescence. Confocal analysis of clonal rat INS-1E β-cells expressing the TRMT10A-hrGFP fusion protein showed nuclear fluorescence with intense signal accumulation in nuclear regions of low Hoechst 33342 staining (Figure 5A). Cells expressing hrGFP alone showed homogeneous cytosolic and nuclear fluorescence. The identity of the recombinant fusion protein expressed in these cells was confirmed by Western blot (Figure 5B) using an antibody raised against purified recombinant hTRMT10A. Similar results were obtained in dispersed rat and human islet cells expressing the recombinant fusion protein (Figure S4). To identify the nuclear compartment enriched in TRMT10A, we performed immunofluorescence in rat and human islet cells using antibodies against hTRMT10A and fibrillarin, a nucleolar marker [23]. Immunostaining of endogenous TRMT10A (Figure 6, red) mimicked the fluorescence profile of recombinant TRMT10A-hrGFP. Fibrillarin immunolabeling showed a similar punctuate nuclear pattern (Figure 6, green). TRMT10A and fibrillarin images were superimposable (Figure 6, merge) indicating that TRMT10A expression is enriched in the nucleolus.

### TRMT10A silencing does not affect insulin secretion but enhances total protein biosynthesis

**Figure 7:**
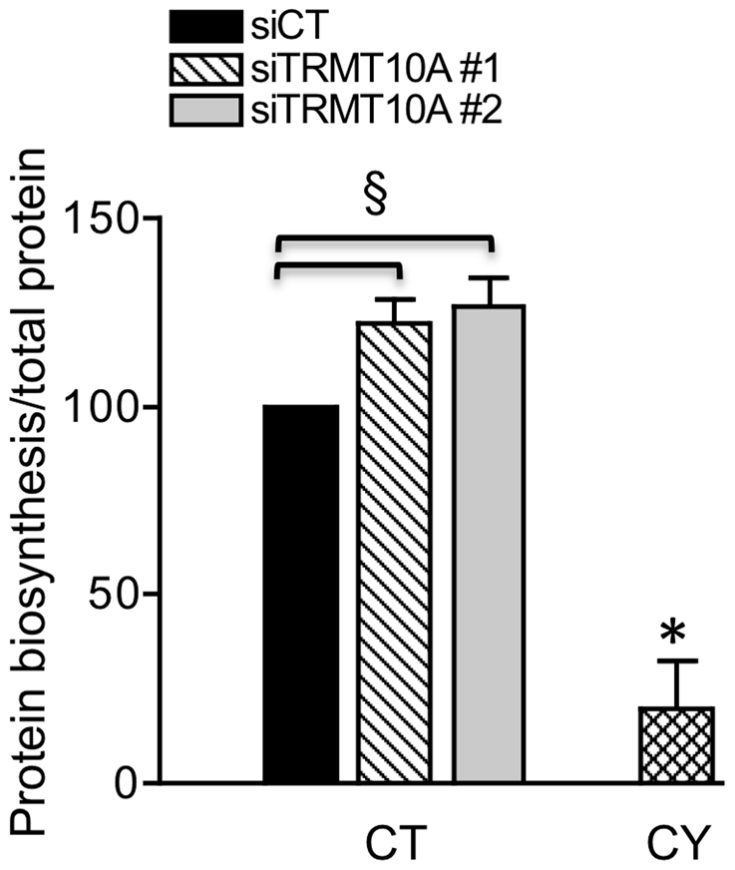
TRMT10A knockdown enhances total protein biosynthesis in rat β-cells. Total protein synthesis was measured in INS-1E cells transfected with control siRNA (siCT) or two siRNAs targeting rat TRMT10A (siTRMT10A #1 and #2). Protein biosynthesis was corrected by total protein content and expressed as % of siCT. INS-1E cells treated for 2 h with the inhibitor of translation cycloheximide (CY, 10 µM) were used as positive control (n = 4). *CY vs CT, § siTRMT10A vs siCT p<0.05 by paired *t* test.

RNA interference technology was used to knock down TRMT10A in β-cells. TRMT10A mRNA and protein expression was reduced by 50% in INS-1E cells (Figure S5). TRMT10A silencing did not modify glucose-induced insulin secretion and insulin content (Figure S6), but enhanced total protein biosynthesis by 25% in clonal rat β-cells (Figure 7).

### TRMT10A silencing sensitizes β-cells to free fatty acid (FFA)- and endoplasmic reticulum (ER) stress-induced apoptosis

**Figure 8:**
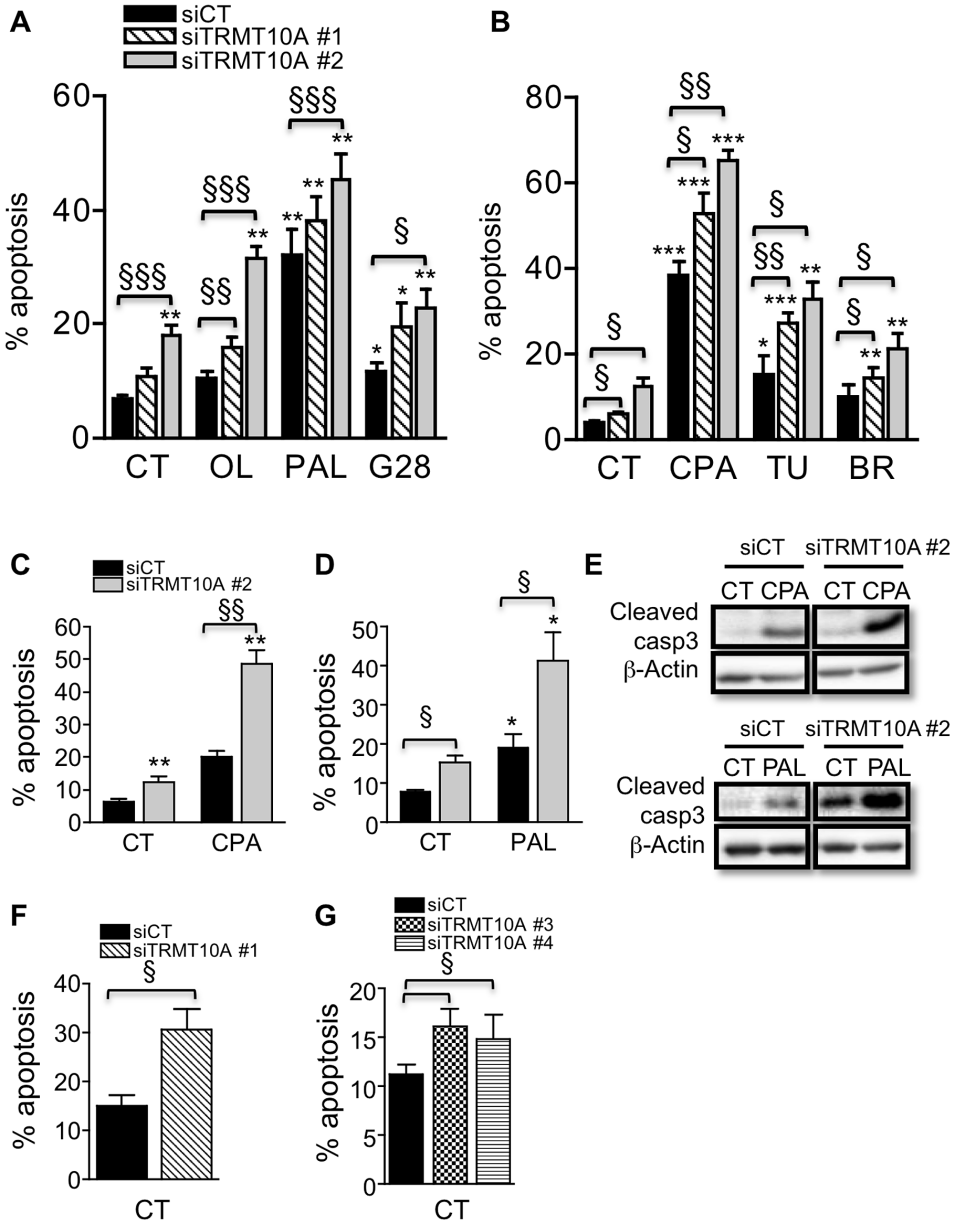
TRMT10A knockdown sensitizes β-cells to FFA-, high glucose- and ER stress-induced apoptosis. INS-1E cells (A–E), primary rat β-cells (F) and dispersed human islets (G) were transfected with control siRNA (siCT) or siRNAs targeting rat (siTRMT10A #1 and #2) or human TRMT10A (siTRMT10A #3 and #4). 48 h after transfection cells were exposed or not (CT) to oleate (OL), palmitate (PAL) and 28 mM glucose (G28), or to the chemical ER stressors cyclopiazonic acid (CPA), tunicamycin (TU) and brefeldin (BR), for 24 (A–B) or 16 h (C–E). Apoptosis was examined by propidium iodide and Hoechst 33342 staining (A–D, F–G) or Western blot for cleaved caspase-3 (E). Results are means ± SE (n = 3–5). The blots are representative of 4 independent experiments. * Treated vs CT; § siTRMT10A vs siCT. One symbol p<0.05, two p<0.01, three p<0.001 by paired *t* test with Bonferroni correction for multiple comparisons.

We next examined whether TRMT10A silencing affects β-cell survival. TRMT10A knockdown induced apoptosis in clonal and primary rat β-cells and dispersed human islets (Figure 8). TRMT10A deficiency further sensitized rat β-cells to oleate-, palmitate- and ER stress-induced apoptosis (Figure 8A–D). These results were confirmed by Western blot for cleaved caspase-3, showing increased caspase-3 activation basally and after palmitate and cyclopiazonic acid exposure (Figure 8E). High glucose-induced β-cell apoptosis was also increased by TRMT10A silencing (Figure 8A). We observed that TRMT10A expression in β-cells is modulated by ER stress. Exposure of rat or human β-cells to the saturated FFA palmitate, previously shown to induce ER stress [3], [24], [25], or to chemical ER stressors enhanced TRMT10A expression (Figure S7) to an extent that was correlated with the intensity of ER stress (measured by the expression of ER stress markers, Figure S8). TRMT10A expression was induced in a PERK- but not IRE1-dependent manner (Figure S9). TRMT10A silencing did not induce expression of the ER stress markers BiP, XBP-1s, ATF3 and CHOP (data not shown).

## Discussion

In a large consanguineous family of Moroccan origin, we identified a new syndrome of severe insulinopenic young onset diabetes and microcephaly with intellectual disability. We used linkage analysis and whole exome sequencing to identify the causal mutation. We found only one region of homozygosity by descent shared by the three affected patients, and only one potentially damaging rare genetic variant in this region, located in the *TRMT10A* gene, changing an arginine codon at position 127 of the protein into a stop codon (Arg127Stop). In the rest of the patients' exome, we found no potentially damaging, rare biallelic variants shared by the three patients that might have qualified for a causal mutation.

Among the family members, four were heterozygous carriers of a mutant allele. Of these, the parents developed diabetes in their fifties, one sister had gestational diabetes, and one brother had normal plasma glucose levels at the age of 23 (Figure 1). Other family members were not available for testing. It is possible that TRMT10A haploinsufficiency increases the risk for adult onset diabetes.

*TRMT10A* contains 8 exons, the first exon being non-protein coding. The mutated codon 127 is in exon 4. The protein environment of Arg127 is extremely conserved across species. Little is known about the role of TRMT10A in mammals. A single study suggested altered TRMT10A mRNA expression in colorectal cancer [26]. Blast analysis indicated that TRMT10A is the mammalian ortholog of *S. cerevisiae* TRM10, previously shown to be involved in guanine 9 tRNA methylation m^1^G_9_ [16]. TRMT10A has seven transcripts in the Vega database. Two of them are non-protein coding due to a retained intron, three contain 8 exons coding for identical proteins of 339 amino acids, and differ only in their untranslated regions. InterProScan analysis indicates that these three proteins have a tRNA (guanine 9-N1) methyltransferase domain as well as tRNA (guanine-N1) methyltransferase domain, both of them present in TRM10. The last two TRMT10A transcripts contain only 6 exons and code for shorter proteins of 200 and 206 amino acids. These two variants are truncated at the C-terminus and only have the tRNA (guanine-N1) methyltransferase domain. In rat only one isoform of TRMT10A containing both domains is found. Based on these analyses, we suggest that TRMT10A functions as a tRNA-modifying enzyme, but this remains to be experimentally confirmed.

The Arg127Stop mutation is predicted to block the expression of the five coding human TRMT10A isoforms. The nonsense mutation abolished TRMT10A protein expression, and also significantly reduced its mRNA expression (Figure 2), probably by nonsense-mediated decay and/or transcriptional silencing [18], [19]. We show that TRMT10A is ubiquitously expressed but enriched in brain and pancreatic islets (Figure 3), consistent with the tissues affected in this new syndrome of diabetes and microcephaly. In silico topology prediction indicates that the five human TRMT10A isoforms, as well as the rat enzyme, have predominant nuclear localization. This was confirmed by immunofluorescence and confocal microscopy, with TRMT10A mainly localizing in the nucleolus of β- and non-β-cells (Figure 5–6 and S4). tRNA transcription and early processing occurs in several subcellular compartments including the nucleus, cytoplasm and cytoplasmic surface of the mitochondria [14]. tRNA genes are recruited to the nucleolus for transcription [27], 5′ leader sequence removal and 3′ end modification, removal of the 3′ trailer and addition of the CCA, which is required for efficient tRNA nuclear export [28]. Mature tRNAs are exported to the cytosol for aminoacylation and function in translation. This transport is not unidirectional; cytosolic tRNAs can follow a retrograde transport to the nucleus (e.g. during nutrient deprivation), to be re-exported to the cytosol following nutrient availability [14]. Some tRNA modifications occur on initial tRNA transcripts, while others are introduced in end-matured tRNAs [29]. Since tRNA transcription and maturation occurs in the nucleus it is expected that the enzymes catalyzing these modifications have a nuclear localization. Studies in yeast confirmed that a subset of tRNA methyltransferases is located in the nucleus [28], [30], [31], with distinct subnuclear distribution, i.e. nucleolus, nucleoplasm, or inner nuclear membrane; the reason for these different localizations is not known [14], [31]. The predominant nucleolar localization of TRMT10A is consistent with its proposed tRNA modifying activity.

Alterations in tRNA modification are expected to affect protein translation. We showed that TRMT10A knockdown in rat β-cells enhances total protein biosynthesis (Figure 7). TRMT10A silencing does not impair glucose-induced insulin secretion or content in β-cells (Figure S6), suggesting that TRMT10A deficiency has no major impact on β-cell function. TRMT10A knockdown sensitizes β-cells to apoptosis in control condition and after exposure to FFAs, high glucose or synthetic ER stressors (Figure 8), conditions related to T2D. It has been proposed that mammalian cytosolic and mitochondrial tRNAs prevent apoptosis by blocking the binding of cytochrome c to Apaf-1, thus preventing the formation of the apoptosome [32], [33]. It is not known whether tRNA modifications affect this tRNA-cytochrome c interaction.

Primary microcephaly refers to a congenitally small but otherwise normally structured brain, with a head circumference later in life that remains 3 SD below the mean for age and gender. Primary microcephaly is a very rare disorder affecting approximately 1/100,000 live births, mainly inherited as an autosomal recessive trait, and is associated with a high rate of parental consanguinity [34]. Microcephaly and young onset diabetes co-segregate in the present family, as both features were present in the three affected siblings and absent in the six unaffected siblings, defining a new syndrome. Our linkage analysis identified a single region where all affected siblings were homozygous over a significant length of genomic DNA. It is hence likely that the whole phenotype results from pleiotropic effects of a single mutated gene.

Microcephaly in our patients was associated with intellectual disability and no other neurological feature, except for a history of petit mal seizures in the proband. This clinical presentation fits with the phenotype of primary microcephaly [35]. Primary microcephaly is vastly heterogeneous, and many genes that cause primary microcephaly play a role in mitotic spindle organization and/or DNA repair, presumably affecting the proliferation of neural progenitors and the generation of an adequate pool of neurons in the developing brain [36].

The expression pattern of TRMT10A in the ventricular zone of the developing cortex is consistent with its influence on neural progenitor properties, including control of survival that is known to affect brain size. In addition it may act in subsets of differentiated neurons, as suggested by its expression in cortical marginal zone and cerebellum.

Early onset diabetes has been associated with microcephaly in other genetic disorders. Homozygous mutations in the *IER3IP1* gene encoding the immediate and early response 3 interacting protein 1 result in infantile diabetes and congenital microcephaly with simplified gyration, hypotonia, intractable seizures, and early death [37], [38]. Cases of microcephaly with severe neurological expression were also described in Wolcott-Rallison syndrome, which includes permanent neonatal diabetes, multiple epiphyseal dysplasia, osteoporosis and liver dysfunction. This syndrome is due to biallelic mutations in *EIF2AK3* encoding translation initiation factor 2-α kinase-3 [39]. EIF2AK3 is activated upon the accumulation of unfolded proteins in the ER and inhibits protein translation initiation [40].

Other human diseases are caused by mutations in genes encoding tRNAs and tRNA modifying enzymes. Pontocerebellar hypoplasia, characterized by hypoplasia and atrophy of ventral pons, cerebellum and the cerebral cortex, is caused by mutations in genes encoding tRNA splicing endonuclease subunits (*TSEN*) or mitochondrial arginyl-tRNA synthetase (*RARS2*) [41]. Mutations in mitochondrial tRNA genes and in aminoacyl-tRNA synthetases cause myopathies and neurodegenerative diseases, sometimes in association with diabetes. Recently, a syndrome of mental retardation, microcephaly and short stature was described, caused by mutations in *NSUN2*, encoding a methyltransferase that catalyzes the intron-dependent formation of 5-methylcytosine at C34 of tRNA-leu(CAA) [42], [43]. NSUN2 is the ortholog of yeast TRM4. Wild-type NSUN2 localized to the nucleolus, whereas mutant NSUN2 accumulated in the nucleoplasm and cytoplasm [42]; other *NSUN2* mutations resulted in nonsense-mediated mRNA decay [43]. Inactivation of the X-linked gene FTSJ1, another RNA methyltransferase and ortholog of yeast TRM7, gives rise to non-syndromic intellectual disability [44].

In addition to causing microcephaly and short stature, the *TRMT10A* mutation causes a severe form of diabetes, which was not reported for these other RNA methyltransferase mutations. This may be related to cell-specific requirements of RNA modifications. It is of particular interest that *CDKAL1* polymorphisms predispose to insulin secretion defects and T2D [8]. CDKAL1 was recently shown to methylthiolate tRNA^Lys^(UUU) [45]. The β-cell-specific Cdkal1 knockout mouse develops impaired glucose tolerance, due to misreading of Lys codons in proinsulin, defective insulin biosynthesis and increased susceptibility to ER stress and high fat diet [9].

In conclusion, we describe a nonsense mutation in the *TRMT10A* gene in a new syndrome of young onset diabetes and microcephaly. Based on its cellular localization and by homology with its yeast counterpart, we propose that TRMT10A has methyltransferase activity. We show that TRMT10A is expressed in human fetal brain; TRMT10A silencing does not impair β-cell function but induces apoptosis, suggesting that TRMT10A deficiency may negatively affect β-cell mass and the pool of neurons in the developing brain. Our findings may have broader relevance for the understanding of the pathogenesis of T2D and microcephaly.

## Materials and Methods

### Ethics statement

The ethics committee of the Erasmus Hospital, Université Libre de Bruxelles approved of the study. The three patients, their parents, and two unaffected siblings gave informed consent. Human fetal brain was collected and used according to the guidelines of the local ethics committees on research involving human subjects (Erasmus Hospital, Université Libre de Bruxelles and Belgian National Fund for Scientific Research). Adult male Wistar rats were housed and used following the rules of the Belgian Regulations for Animal Care, with approval of the ethics committee of the Université Libre de Bruxelles.

### Linkage analysis

A peripheral blood sample was obtained for genetic analysis from the three patients, their parents, and two unaffected siblings. Leukocyte DNA was extracted using proteinase K digestion followed by phenol-chloroform extraction and ethanol precipitation [46] and samples were stored at 4°C in T10E1 buffer. We used Affymetrix 11K-GeneChip microarrays representing 10,000 autosomal single nucleotide polymorphisms (Affymetrix, High Wycombe, United Kingdom) to genotype the three patients' DNA (500 ng each) on an Affymetrix platform following the instructions of the manufacturer. Regions of homozygosity were delineated using the ExcludeAR algorithm [47]. In chromosomal regions with apparent homozygosity by descent, microsatellites were genotyped in individual subjects. Marker order was obtained from the University of California at Santa Cruz (UCSC) physical map (http://genome.ucsc.edu/cgi-bin/hgGateway). A multipoint LOD score was computed using the MAPMAKER/HOMOZ software [48] assuming a gene frequency of 0.005 and marker allele frequencies as observed in a series of control subjects, with a minimal minor allele frequency of 0.10.

### Whole exome sequencing

Genomic DNA from the proband (Figure 1, arrow) was sonicated and enriched for exonic sequences by hybridization on an Agilent SureSelect All Exon v1 capture kit. Exon-enriched DNA was paired-end sequenced over 90 bp by an Illumina HiSeq2000 sequencer (Beijing Genomics Institute). An average of 55.6 million paired-end reads were filtered to eliminate reads with more than 6 undetermined nucleotides or 40 identical bases in tandem. The filtered reads were then aligned to the human genome GRCh36 assembly using the SOAPaligner 2.20 software [49] and the genotypes were called using the SOAPsnp program [50]. Resulting single nucleotide variants (SNVs) were filtered according to the following rules: base quality larger than 20, read depth equal to or larger than 4, and a distance between two variants larger than 4. Insertions and deletions were identified separately, through alignment to GRCh36 using the Burrows-Wheeler alignment tool [51], and detection using the Genome Analysis Toolkit [52]. SNVs and indels were annotated using the Ensembl V54 database. We considered SNVs and indels that were not found in the dbSNP135 database, nor in the Thousand Genome (www.1000genomes.org) database, nor in the Exome Variant Server (http://evs.gs.washington.edu/EVS/), and that were not found in our other in-house exome sequencing results.

### Sanger sequencing

PCR primers for all exons and flanking intronic sequences were designed using the Exonprimer software (http://ihg.helmholtz-muenchen.de/ihg/ExonPrimer.html). All exons and flanking intronic regions of the candidate genes were sequenced by the Sanger method using the Big Dye Terminator cycle sequencing kit v2 (Applied Biosystems, Foster City, California, USA), and analyzed on a 3130 Genetic Analyser sequencing machine (Applied Biosystems). Sequences were analyzed in silico for mutations using the SeqScape software V.2.0. (Applied Biosystems).

### RNA in situ hybridization

*In situ* hybridization was done on human fetal brain (GW 8,11, 17, 19) as previously described [53]. Riboprobe template was generated by PCR using TRMT10A specific pairs of primers: F: CCAAGCTAATACGACTCACTATAGGGAGATGTGAACCAATATCTAAACGACAAA – R: GGATCCATTAACCCTCACTAAAGGGAGAGATTTTCCTTATCCTGCTTTTCTTC.

### Culture of INS-1E cells, FACS-purified primary rat β-cells, human islets and human lymphoblasts

Clonal rat INS-1E cells (a kind gift from Dr C Wollheim, Centre Médical Universitaire, Geneva, Switzerland) were cultured in RPMI medium as previously described [54], [55]. Tissues were obtained from adult male Wistar rats (Charles River Laboratories). Rat islets were isolated by collagenase digestion followed by hand picking under a stereomicroscope. Islets were dispersed and β-cells purified by autofluorescence-activated cell sorting (FACS, FACSAria, BD Bioscience) and cultured as described [56], [57]. Human islets from non-diabetic organ donors (n = 13, age 68±4 years, BMI 27±1 kg/m^2^) were isolated by collagenase digestion and density gradient purification [58]. The islets were cultured, dispersed and transfected as previously described [59]. The mean percentage of β-cells of the human islet preparations was 50±5%, as determined by insulin immunofluorescence [25], [60]. Human lymphoblasts from three control individuals, two patients and one heterozygous carrier of the mutation were cultured in RPMI 1640 medium supplemented with 20% FBS, 100 mU/ml penicillin and 100 mU/ml streptomycin.

### Recombinant human TRMT10A: cloning, expression, purification and antibody production

hTRMT10A was amplified by PCR from lymphoblast cDNA using oligonucleotides spanning the start and stop codons of the *TRMT10A* open reading frame (ORF), using primers F CGGAATTCATGTCATCTGAAATGTTGCC and R CGCTCGAGGTGTGGCAGAGAGTTCACTG. The restriction sites *EcoR*I and *Xho*I (underlined) were added to facilitate the directional cloning into the expression vector pGEX-6P-1 (GE Healthcare). This vector allows the expression of recombinant proteins fused to glutathione-s-transferase (GST) at its N-terminus. *E. coli* BL21 cells were transformed with the pGEX-6P-1-TRMT10A plasmid by electroporation. Positive clones were selected by colony PCR and sequenced. For recombinant protein expression, a single colony was grown overnight at 37°C in LB medium containing 100 µg/ml ampicillin. Cells were then diluted 1∶50 in the same medium and grown at 37°C until an optical density of 0.6 at 600 nm was reached. Isopropyl-β-D-thiogalactoside (0.25 mM) was then added and cells were grown at 28°C for 3 h to induce recombinant protein expression. Cells were harvested by centrifugation at 3000×g for 10 min, lysed by sonication in 20 mM Tris buffer pH 8 containing 0.5% Triton ×100, 10 mM dithiothreitol, 0.1 mM PMSF and protease inhibitor cocktail (Roche), and centrifuged for 15 min at 15,000×g at 4°C. The supernatant was applied to 1 ml glutathione spin columns (Pierce) and washed with ice-cold lysis buffer. The recombinant hTRMT10A was separated from the GST moiety by *in column* site-specific proteolysis using PreScission protease (GE Healthcare) following the manufacturer's instructions. The purified recombinant hTRMT10A was used for rabbit polyclonal antibody production (Eurogentec).

### Recombinant human TRMT10A-hGFP

hTRMT10A was amplified by PCR from HeLa cDNA using the oligonucleotides F 5′-AAAAAACCCGGGA**ATG**TCATCTGAAATGTTG-3′ (start codon is indicated in bold), and R 5′-AAAAAAGGATCCTGAGTGTGGCAGAGAGTT-3′ in which the restriction sites *Sma*I and *Bam*HI (underlined) were added to facilitate the directional cloning into the mammalian expression vector Vitality phrGFP-1 (Stratagene). The stop codon of the TRMT10A ORF was removed to allow the production of the recombinant TRMT10A fused to the N-terminus of hrGFP. The PCR product was purified using the Wizard SV Gel and PCR clean-up system (Promega), sequentially digested with *Sma*I and *Bam*HI (New England Biolabs), and cloned into the Vitality phrGFP-1 vector digested with the same restriction enzymes. The plasmid was introduced into electrocompetent One Shot *E. coli* (Invitrogen) by electroporation, and positive clones were identified by colony PCR. A single colony containing hrGFP (empty vector) or TRMT10A-hrGFP plasmid was grown overnight at 37°C in LB medium with 100 µg/ml ampicillin. Plasmids were purified with PureYield Midiprep (Promega) and quantified by NanoDrop (Thermo Scientific). Expression of recombinant TRMT10A-hrGFP in rat β-cells was confirmed by Western blot.

### RNA interference and plasmid transfections

Cells were transfected overnight with 30 nM of a control siRNA (Qiagen), or two single siRNAs targeting rat or human TRMT10A using Lipofectamine RNAiMAX (Invitrogen). siRNA-lipid complexes were formed in Opti-mem (Invitrogen) for 20 min as previously described [61].

hrGFP and TRMT10A-hrGFP plasmids were introduced by lipofection in INS-1E cells or dispersed rat and human islet cells using Lipofectamine 2000 (Invitrogen). siRNA sequences, plasmid and Lipofectamine concentrations are described in Tables S1 and S2.

### Immunofluorescence and confocal microscopy

TRMT10A subcellular localization was examined by expressing recombinant hTRMT10A fused to GFP, or by immunolabeling endogenous TRMT10A in INS-1E cells and dispersed rat and human islet cells. Cells plated on poly-lysine coated cover slips were transfected or not with hrGFP and TRMT10A-hrGFP plasmids, fixed with 4% formaldehyde [62], permeabilized with methanol, blocked with goat serum and incubated or not for 1 h with rabbit anti-hTRMT10A (1∶200, Eurogentec), mouse anti-human/rat fibrillarin (1∶200, EnCor Biotechnology) and mouse anti human/rat insulin (Sigma). Alexa Fluor 546 goat anti-mouse IgG (H+L), Alexa Fluor 488 goat anti-mouse IgG (H+L) and Alexa Fluor 546 goat anti-rabbit IgG (H+L) (1∶500, Molecular Probes, Invitrogen) were used as secondary antibodies. Nuclei were stained with Hoechst 33342. Slides were analyzed by inverted fluorescence microscopy (Zeiss Axiovert 200, Oberkochen, Germany). Confocal analysis was performed on a LSM510 NLO multiphoton confocal microscope fitted on an Axiovert M200 (Zeiss) [63].

### Total RNA and mRNA extraction and real-time PCR

Poly(A)^+^ mRNA was isolated from INS-1E cells, dispersed human islets, or primary rat β-cells using the Dynabeads mRNA DIRECT kit (Invitrogen). For total RNA purification, rat tissues and pancreatic islets were resuspended in RNeasy Minikit lysis buffer (Qiagen), homogenized using a T10 basic ULTRA-TURRAX disperser (IKA) or lysed by sonication in a Bioruptor NGS (Diagenode), respectively. Total RNA was purified with the RNeasy Minikit and quantified by NanoDrop. mRNA and total RNA were reverse transcribed as previously described [25], [56]. Real-time PCR was performed using Rotor-Gene SyBR Green on a Rotor-Gene Q cycler (Qiagen), or FastStart SYBR Green on the LightCycler (Roche Diagnostics) [62], [64]. Standards were prepared using suitable primers in a conventional PCR. Gene expression was calculated as copies/µl using the standard curve approach [65]. Expression values were corrected for the expression of the reference genes GAPDH, OAZ1 and/or β-actin, which were not modified by the experimental conditions. The primers are provided in Table S3.

### Western blotting

Rat tissues and pancreatic islets were resuspended in ice-cold PBS containing protease inhibitor cocktail and homogenized as described above. Total protein was measured in the lysates using the Protein Assay Dye Reagent (BIO-RAD). INS-1E cells and human lymphoblasts were lysed with Laemmli buffer [59]. Cell lysates were resolved in 10 or 14% SDS-PAGE gels and transferred to nitrocellulose membranes. Immunoblotting was performed using antibodies against hTRMT10A, human cleaved caspase-3 (Cell Signaling), human α-tubulin (Sigma-Aldrich) or human β-actin (Cell Signaling). Protein detection was done using horseradish peroxidase-conjugated secondary antibodies and SuperSignal West Femto chemiluminescence revealing reagent (Thermo Scientific). Immunoreactive bands were detected with a ChemiDoc XRS+ system and with Image Lab software (BIO-RAD). Protein levels were corrected for α-tubulin and/or β-actin.

### Glucose-stimulated insulin secretion

Insulin secretion was performed as previously described [61]. Briefly, 72 h after transfection, INS-1E cells were cultured for 1 h in RPMI without glucose, washed with modified Krebs-Ringer bicarbonate HEPES solution, and insulin secretion was induced by 30 min incubation with KRBH containing 1.67 or 16.7 mM glucose, alone or in combination with 10 µM forskolin. Insulin was measured by ELISA (Mercodia) in cell-free supernatants and acid-ethanol extracted cell lysates [61], [66], [67]. Total protein was measured in cell lysates as described above.

### Cell treatment and apoptosis assays

FFA treatment was performed in RPMI 1640 containing 0.75% FFA-free BSA (Roche). Oleate and palmitate (sodium salt, Sigma) were dissolved in 90% ethanol and diluted 1∶100 to a final concentration of 0.5 mM [25], [68]. The chemical ER stressors cyclopiazonic acid and thapsigargin (two SERCA pump blockers), tunicamycin (an inhibitor of N-glycosylation) and brefeldin-A (an inhibitor of ER-to-Golgi vesicle transport) were used at 25 µM, 1 µM, 5 µg/ml and 0.1 µg/ml, respectively. The IRE1 inhibitor 4μ8C was used at 25 µM [69] For all treatments the control condition contained the same dilution of vehicle.

Apoptotic cell death was detected and counted by fluorescence microscopy after Hoechst 33342 (5 µg/ml; Sigma-Aldrich) and propidium iodide (5 µg/ml) staining as described [25], [60], [62], [70]. Apoptosis was also examined by Western blotting for cleaved caspase-3.

### Total protein biosynthesis

72 h after transfection INS-1E cells were cultured for 2 h in Krebs-Ringer buffer containing 11 mM glucose, 1% BSA and 10 µCi/ml L-(3,4,5 ^3^H)-leucine (Perkin Elmer). Cells were then washed with Krebs-Ringer solution containing 10 mM non-radioactive leucine. Cells were collected in ice-cold water and lysed by sonication. Total protein was precipitated with 10% trichloro-acetic acid. The content of ^3^H-labeled proteins was determined in a liquid scintillation analyzer (Packard) [70]. Protein biosynthesis was expressed per total protein content to correct for differences in cell number in the experimental conditions.

### Statistical analysis

Data are presented as means ± SE. Non-normally distributed variables were log-transformed before statistical testing. Comparisons between groups were made by ANOVA followed by two-sided Student's paired *t* test with Bonferroni correction for multiple comparisons. A p value<0.05 was considered statistically significant.

## Supporting Information

Figure S1 Linkage analysis. Single Nucleotide Polymorphisms rs4128340 and rs10516462 are located at 88,908,073 and 101,307,637 respectively on chromosome 4, GRCh37/hg19 assembly. Microsatellite D4S1628 is at 98,286,500. Homozygosity for the 15 contiguous microsatellites, as well as for 288 additional SNPs (vertical bar, individual SNPs not shown) is observed in the three affected siblings. A critical recombination event (boxed) is observed distal to D4S1628 in an unaffected brother, who is otherwise homozygous for all microsatellite markers. The TRMT10A gene (arrowhead) is at chr4:100,467,864–100,485,189.
(TIF)

Figure S2 Species comparison shows very high conservation of TRMT10A Arginine 127 and surrounding amino acids.
(TIF)

Figure S3 TRMT10A expression profile in fetal brain at 19 GW. (A) Cresyl Violet. (B) TRMT10A antisense (AS) probe showing expression in the presumptive cerebellar cortex. (C) TRMT10A sense (S) probe as a negative control.
(TIF)

Figure S4 TRMT10A has a nucleolar localization in islet cells. Dispersed rat (A) and human islet cells (B–C) were transfected with a vector encoding hrGFP alone (hrGFP) or fused to TRMT10A (TRMT10A-hrGFP). 48 h after transfection TRMT10A subcellular localization was examined by fluorescence microscopy. Nuclei were stained with Hoechst 33342. Nucleolus was stained with anti-fibrillarin antibody. Pictures were taken at 40× magnification and are representative of two independent experiments.
(TIF)

Figure S5 TRMT10A knockdown in INS-1E cells. INS-1E cells were transfected with control siRNA (siCT) or two siRNAs targeting rat TRMT10A (siTRMT10A #1 and #2). 72 h after transfection TRMT10A mRNA and protein expression was examined by real-time PCR and Western blot. (A) TRMT10A mRNA expression corrected for the reference gene GAPDH. (B) Representative Western blot and (C) densitometry of TRMT10A protein expression corrected by α-tubulin or β-actin. Data are means ± SE (n = 4). § p<0.05 by paired t test.
(TIF)

Figure S6 TRMT10A knockdown does not modify insulin secretion or content in β-cells. INS-1E cells were transfected with control siRNA (siCT) or two siRNAs targeting rat TRMT10A (siTRMT10A #1 and #2). (A) 72 h after transfection insulin secretion was induced by 1.67 or 16.7 mM glucose or 16.7 mM glucose +10 µM forskolin (FK). (B) Insulin content corrected by total protein. Results are means ± SE (n = 4). * 16 mM or 16 mM + FK vs 1.67 mM glucose. One symbol p<0.05, two p<0.01, by ratio *t* test.
(TIF)

Figure S7 Endoplasmic reticulum stress enhances TRMT10A expression in INS-1E cells and human islets. TRMT10A mRNA expression was examined by real-time PCR in INS-1E cells and human islets exposed or not (CT) to oleate (OL), palmitate (PAL), 28 mM glucose (G28), CPA, thapsigargin (THAP), tunicamycin (TU) or brefeldin (BR). (A) Time course of TRMT10A mRNA expression in FFA-treated INS-1E cells (n = 3–4). Data is expressed as fold of untreated control. (B–C) INS-1E cells exposed for 24 h to FFAs, high glucose or synthetic ER stressors. Data was normalized to the geometric mean of GAPDH, tubulin and OAZ1 mRNA expression (n = 4–11). (D) Human islets exposed for 24 h to synthetic ER stressors. Expression data was normalized to β-actin (n = 5). * Treated vs CT. One symbol p<0.05, two p<0.01, three p<0.001, by paired *t* test.
(TIF)

Figure S8 Expression of ER stress markers after exposure to synthetic ER stressors. XBP-1s, CHOP, BiP and ATF-3 mRNA expression was examined in INS-1E cells and human islets exposed or not (CT) to CPA, thapsigargin (THAP), tunicamycin (TU) or brefeldin (BR). Expression was normalized to GAPDH or β-actin (n = 4–5). * Treated vs CT. One symbol p<0.05, two p<0.01, three p<0.001, by ratio *t* test.
(TIF)

Figure S9 Endoplasmic reticulum stress modulates TRMT10A expression via PERK but not IRE1 activation. (A–C) INS-1E cells were transfected with control siRNA (siCT), or siRNA targeting rat PERK. 48 h after transfection cells were exposed or not to palmitate (PAL) or CPA for 16 h. (D–F) INS-1E cells were exposed for 16 h to palmitate (PAL) or CPA alone or combined with the IRE1 inhibitor 4μ8C. TRMT10A, ATF3 and XBP-1s mRNA expression was examined by real-time PCR and normalized to GAPDH expression (n = 4–6). * Treated vs CT, § siPERK vs siCT, ## IRE1 inhibitor vs CT. One symbol p<0.05, two p<0.01, three p<0.001, by ratio *t* test.
(TIF)

Table S1 Sequence of rat and human siRNAs.
(DOCX)

Table S2 Transfection reagents used in INS-1E cells, primary rat β-cells and human islets.
(DOCX)

Table S3 5′-3′ primer sequence used for standard and real-time PCR.
(DOCX)
