# Supplementary figures and images for: tRNA Methyltransferase Homolog Gene TRMT10A Mutation in Young Onset Diabetes and Primary Microcephaly in Humans
Source: PLoS Genet. 2013 Oct 31;9(10):e1003888. doi: 10.1371/journal.pgen.1003888 (PMC3814312; doi:10.1371/journal.pgen.1003888)

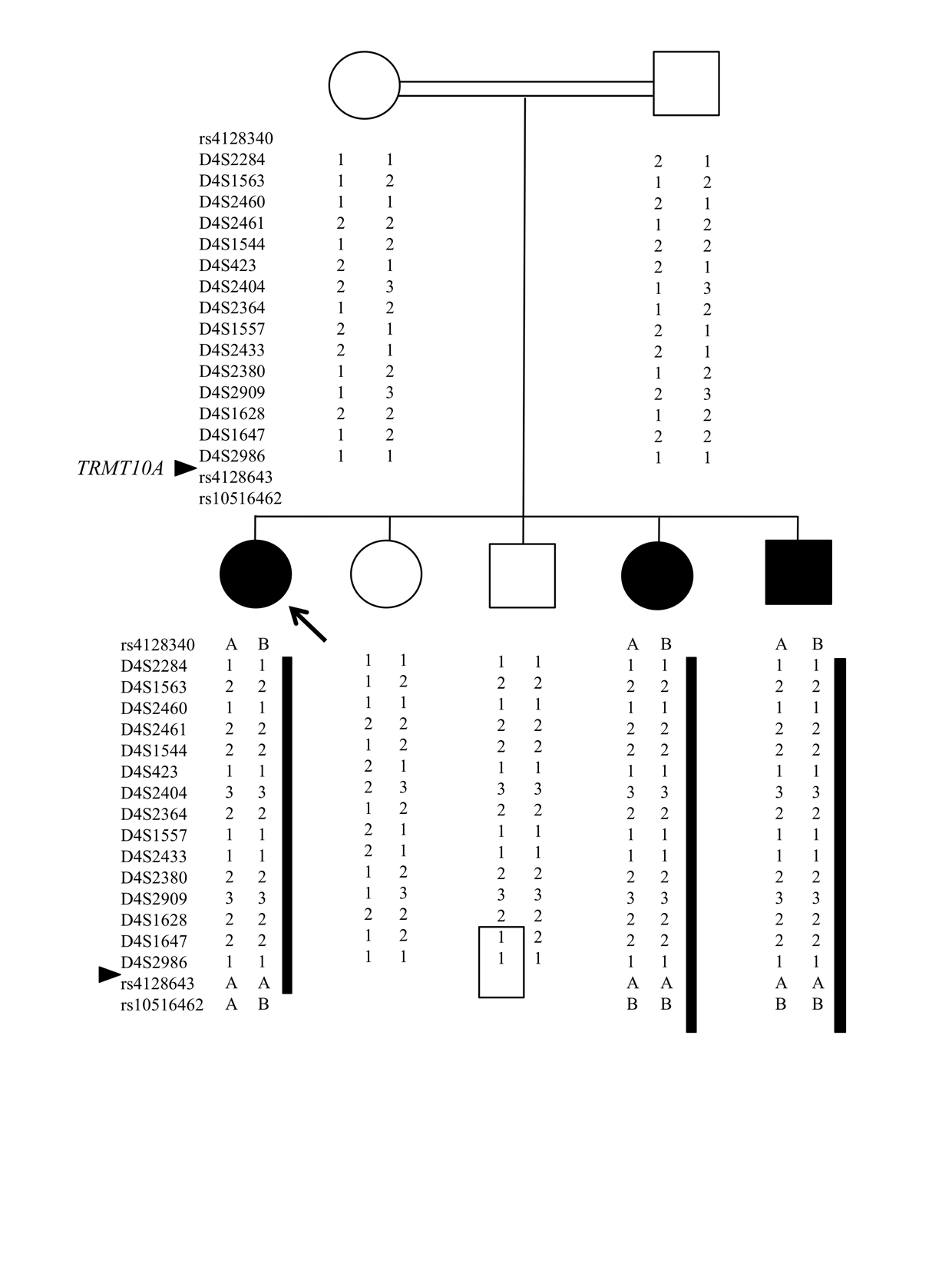

Supplement: Figure S1 — Linkage analysis. Single Nucleotide Polymorphisms rs4128340 and rs10516462 are located at 88,908,073 and 101,307,637 respectively on chromosome 4, GRCh37/hg19 assembly. Microsatellite D4S1628 is at 98,286,500. Homozygosity for the 15 contiguous microsatellites, as well as for 288 additional SNPs (vertical bar, individual SNPs not shown) is observed in the three affected siblings. A critical recombination event (boxed) is observed distal to D4S1628 in an unaffected brother, who is otherwise homozygous for all microsatellite markers. The TRMT10A gene (arrowhead) is at chr4:100,467,864–100,485,189. (TIF) [file pgen.1003888.s001.tif]

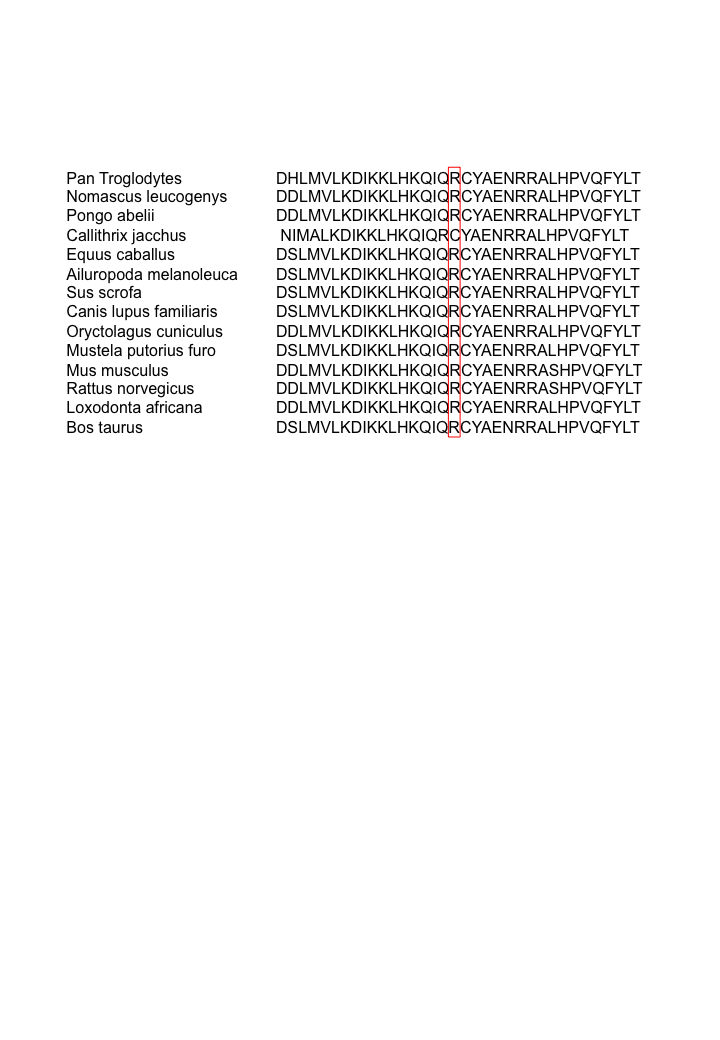

Supplement: Figure S2 — Species comparison shows very high conservation of TRMT10A Arginine 127 and surrounding amino acids. (TIF) [file pgen.1003888.s002.tif]

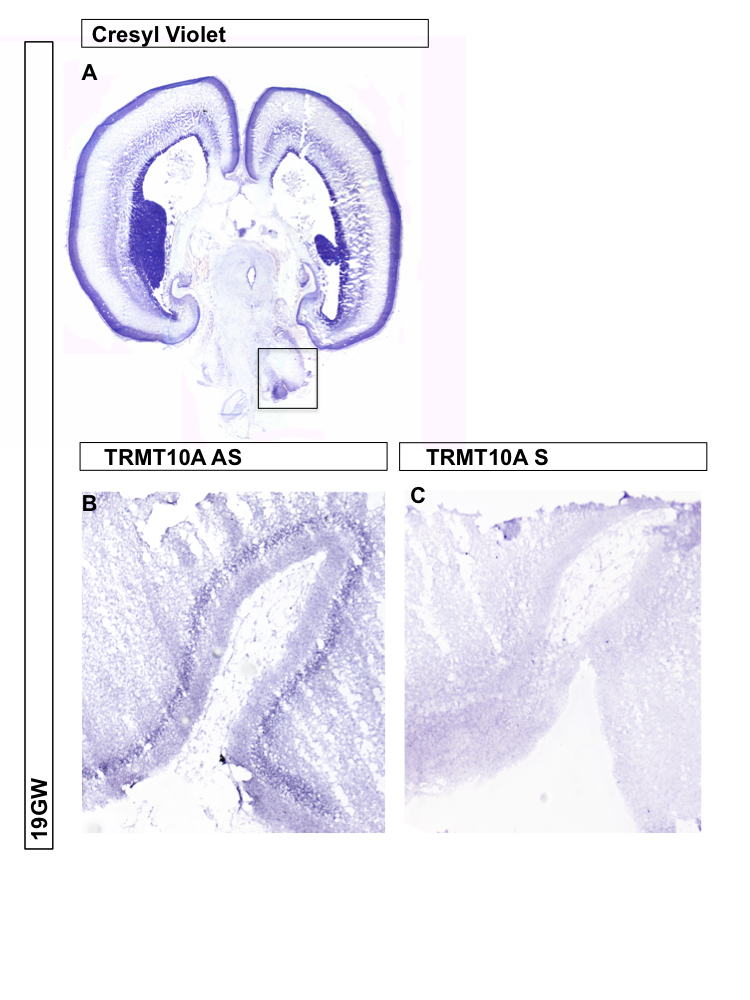

Supplement: Figure S3 — TRMT10A expression profile in fetal brain at 19 GW. (A) Cresyl Violet. (B) TRMT10A antisense (AS) probe showing expression in the presumptive cerebellar cortex. (C) TRMT10A sense (S) probe as a negative control. (TIF) [file pgen.1003888.s003.tif]

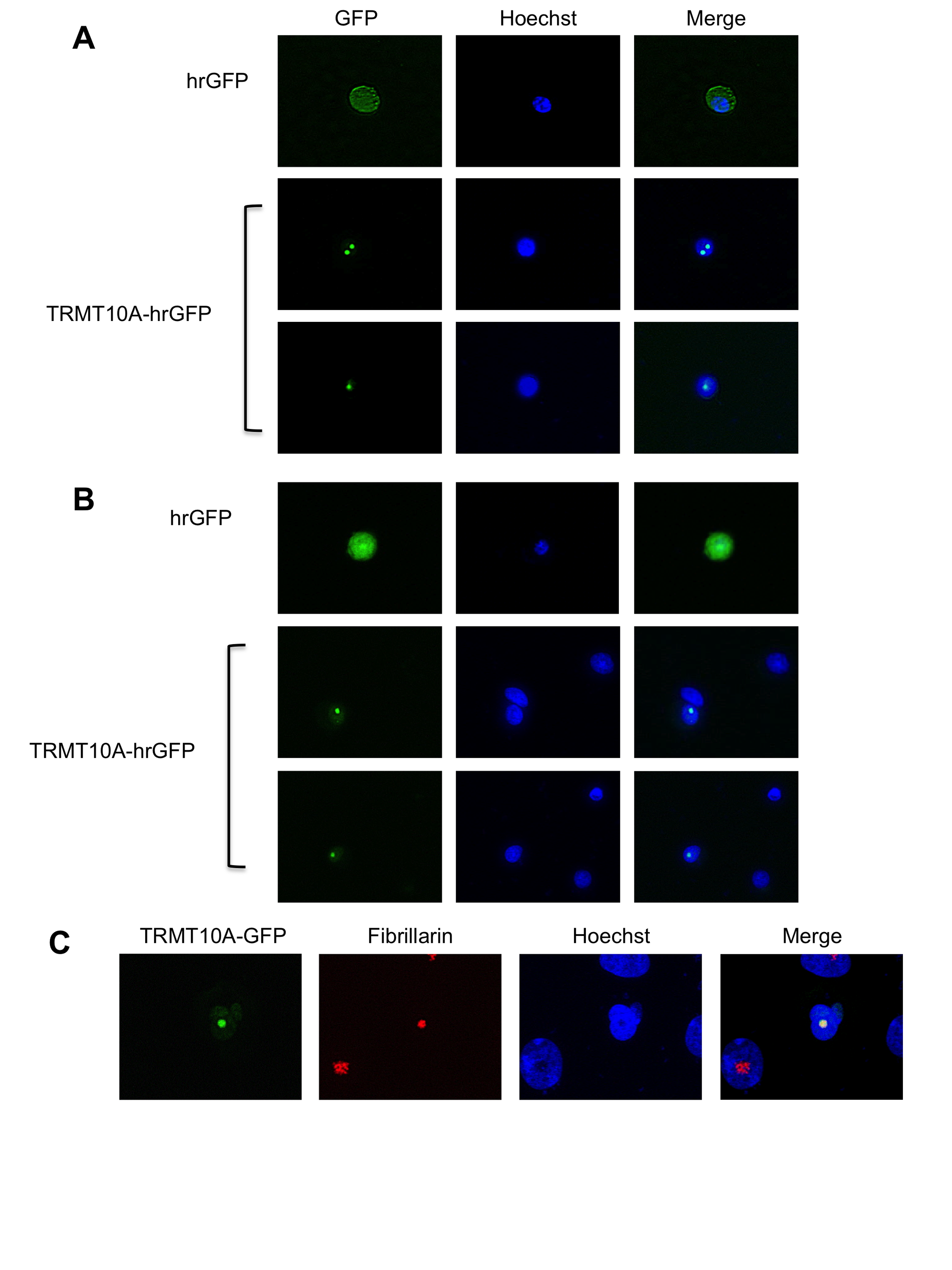

Supplement: Figure S4 — TRMT10A has a nucleolar localization in islet cells. Dispersed rat (A) and human islet cells (B–C) were transfected with a vector encoding hrGFP alone (hrGFP) or fused to TRMT10A (TRMT10A-hrGFP). 48 h after transfection TRMT10A subcellular localization was examined by fluorescence microscopy. Nuclei were stained with Hoechst 33342. Nucleolus was stained with anti-fibrillarin antibody. Pictures were taken at 40× magnification and are representative of two independent experiments. (TIF) [file pgen.1003888.s004.tif]

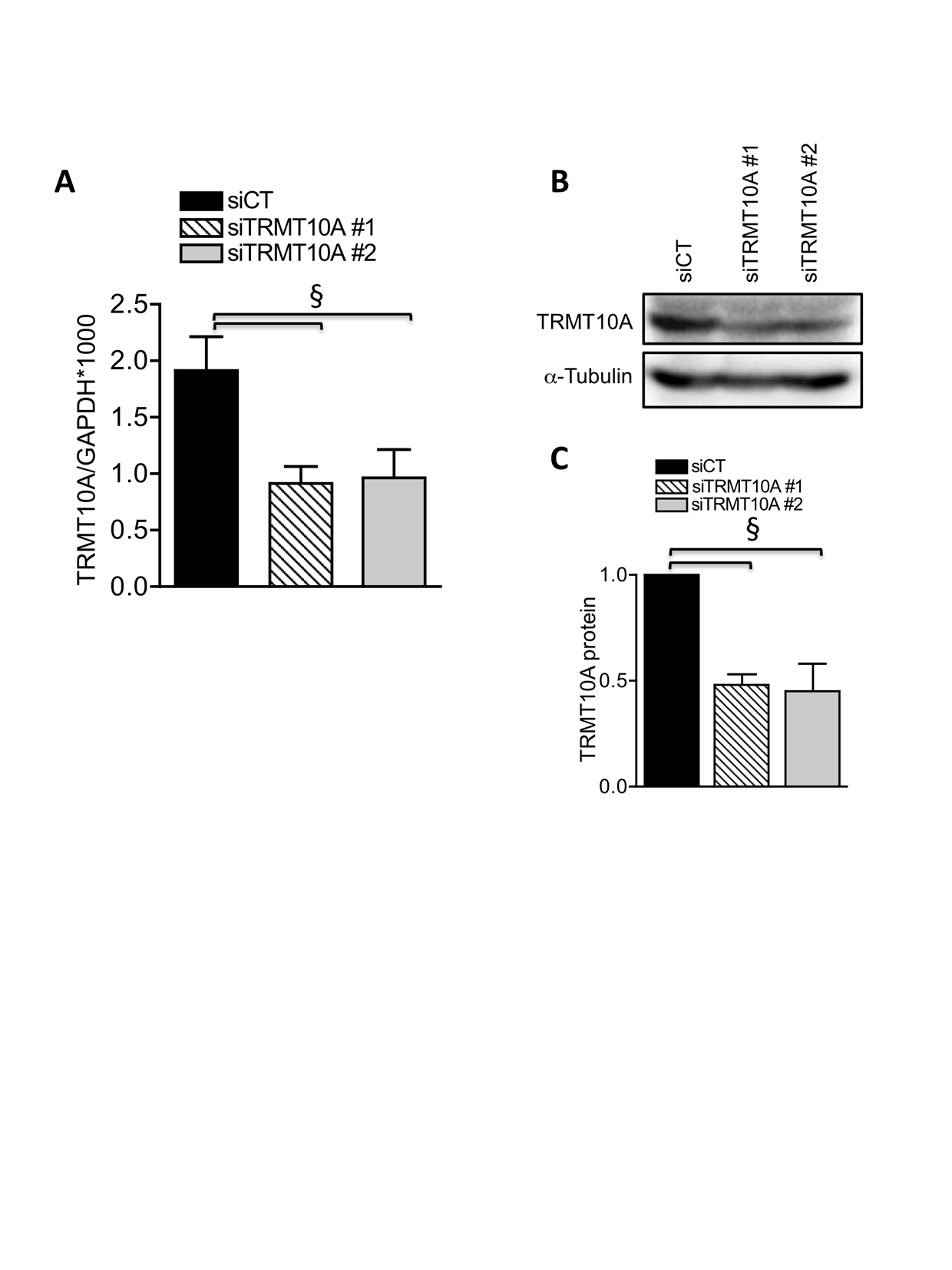

Supplement: Figure S5 — TRMT10A knockdown in INS-1E cells. INS-1E cells were transfected with control siRNA (siCT) or two siRNAs targeting rat TRMT10A (siTRMT10A #1 and #2). 72 h after transfection TRMT10A mRNA and protein expression was examined by real-time PCR and Western blot. (A) TRMT10A mRNA expression corrected for the reference gene GAPDH. (B) Representative Western blot and (C) densitometry of TRMT10A protein expression corrected by α-tubulin or β-actin. Data are means ± SE (n = 4). § p<0.05 by paired t test. (TIF) [file pgen.1003888.s005.tif]

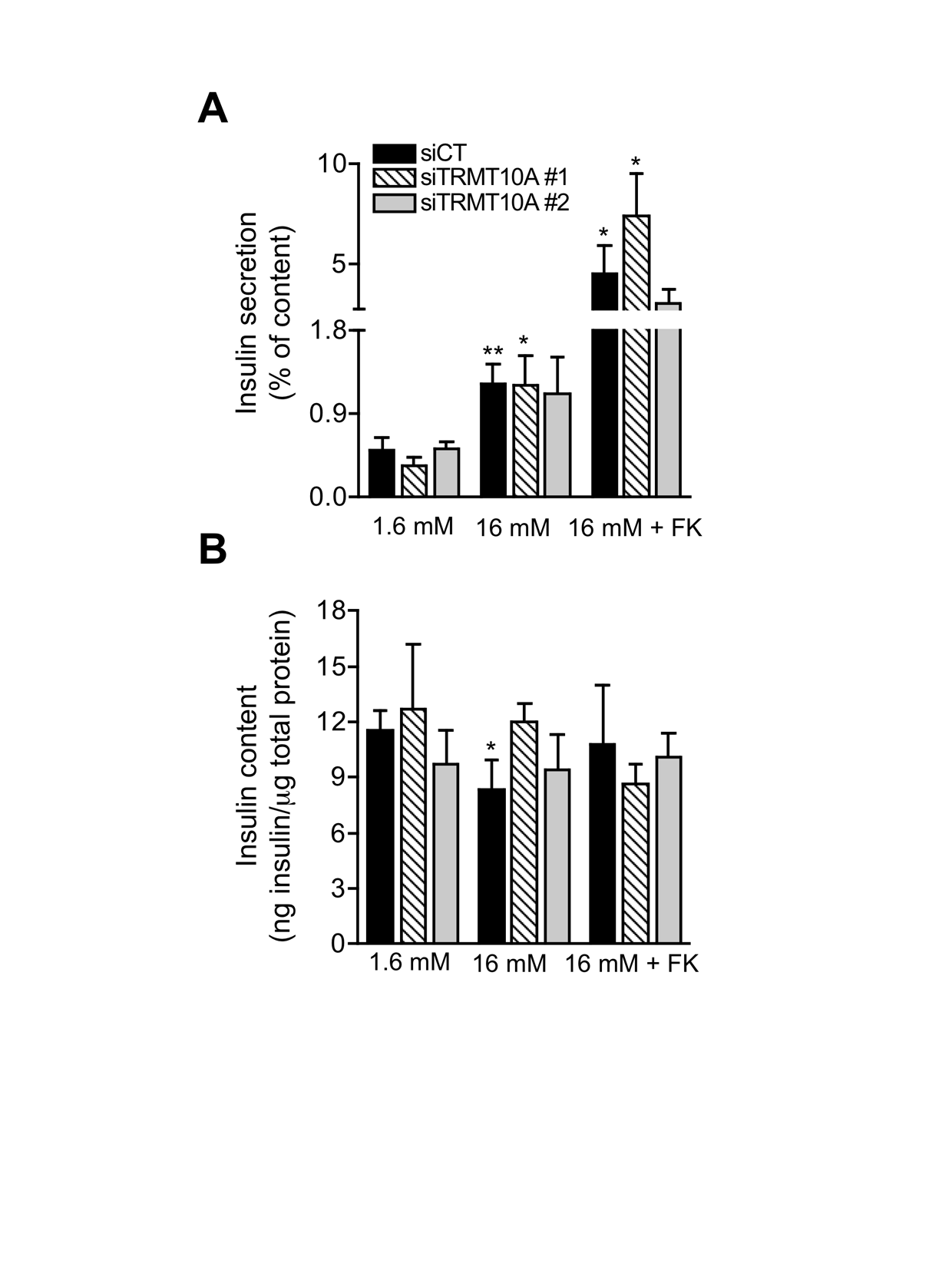

Supplement: Figure S6 — TRMT10A knockdown does not modify insulin secretion or content in β-cells. INS-1E cells were transfected with control siRNA (siCT) or two siRNAs targeting rat TRMT10A (siTRMT10A #1 and #2). (A) 72 h after transfection insulin secretion was induced by 1.67 or 16.7 mM glucose or 16.7 mM glucose +10 µM forskolin (FK). (B) Insulin content corrected by total protein. Results are means ± SE (n = 4). * 16 mM or 16 mM + FK vs 1.67 mM glucose. One symbol p<0.05, two p<0.01, by ratio t test. (TIF) [file pgen.1003888.s006.tif]

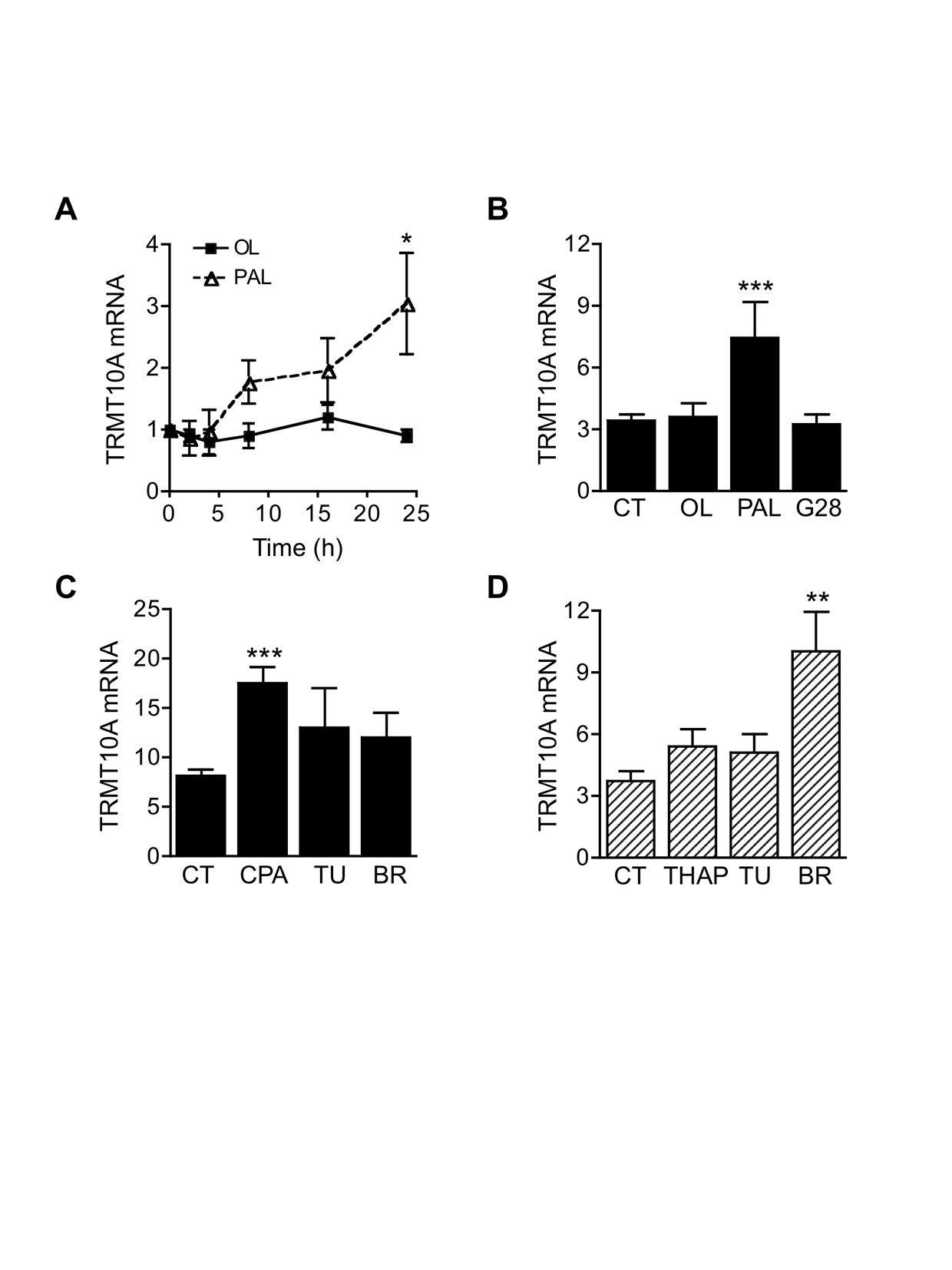

Supplement: Figure S7 — Endoplasmic reticulum stress enhances TRMT10A expression in INS-1E cells and human islets. TRMT10A mRNA expression was examined by real-time PCR in INS-1E cells and human islets exposed or not (CT) to oleate (OL), palmitate (PAL), 28 mM glucose (G28), CPA, thapsigargin (THAP), tunicamycin (TU) or brefeldin (BR). (A) Time course of TRMT10A mRNA expression in FFA-treated INS-1E cells (n = 3–4). Data is expressed as fold of untreated control. (B–C) INS-1E cells exposed for 24 h to FFAs, high glucose or synthetic ER stressors. Data was normalized to the geometric mean of GAPDH, tubulin and OAZ1 mRNA expression (n = 4–11). (D) Human islets exposed for 24 h to synthetic ER stressors. Expression data was normalized to β-actin (n = 5). * Treated vs CT. One symbol p<0.05, two p<0.01, three p<0.001, by paired t test. (TIF) [file pgen.1003888.s007.tif]

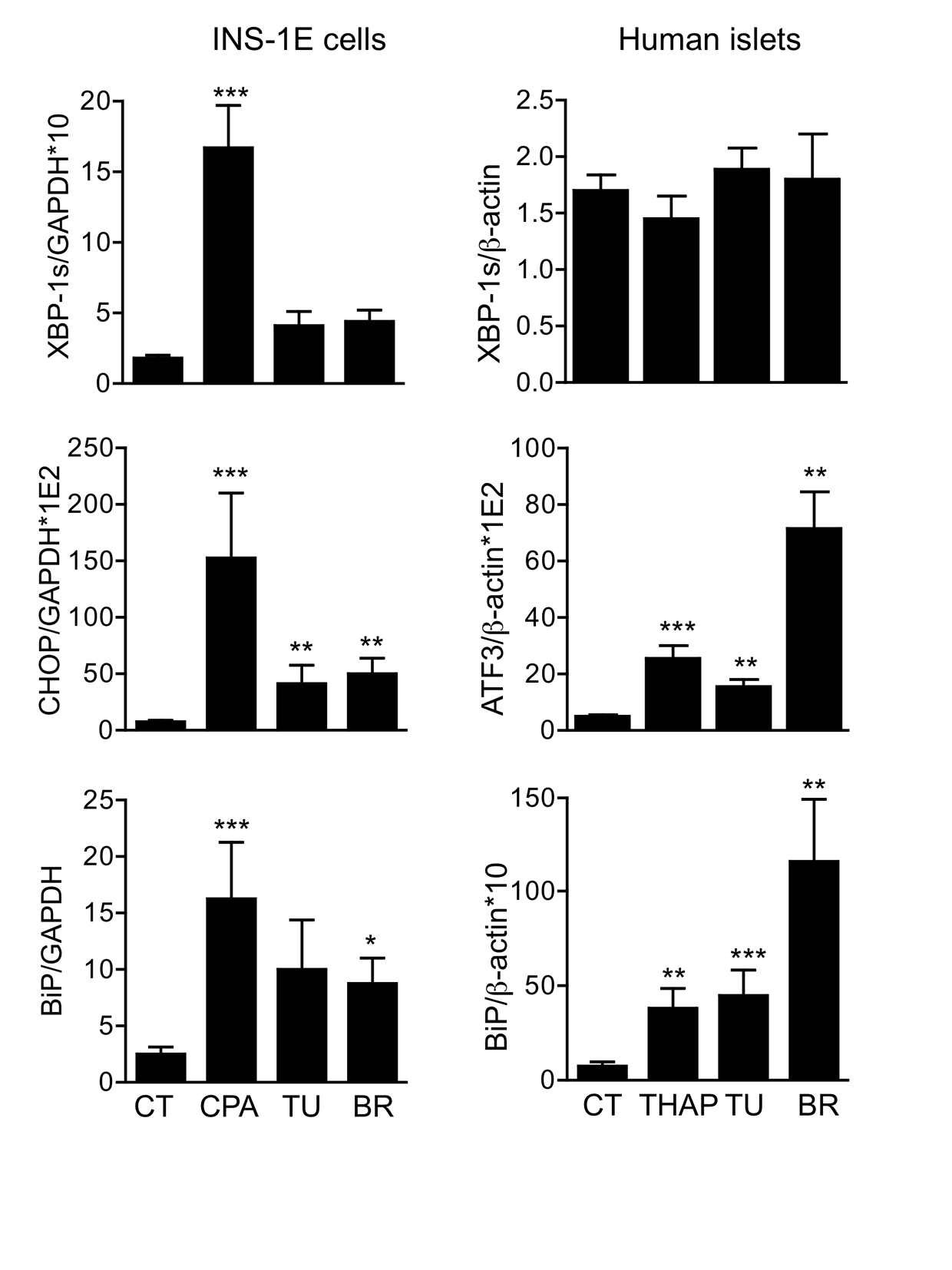

Supplement: Figure S8 — Expression of ER stress markers after exposure to synthetic ER stressors. XBP-1s, CHOP, BiP and ATF-3 mRNA expression was examined in INS-1E cells and human islets exposed or not (CT) to CPA, thapsigargin (THAP), tunicamycin (TU) or brefeldin (BR). Expression was normalized to GAPDH or β-actin (n = 4–5). * Treated vs CT. One symbol p<0.05, two p<0.01, three p<0.001, by ratio t test. (TIF) [file pgen.1003888.s008.tif]

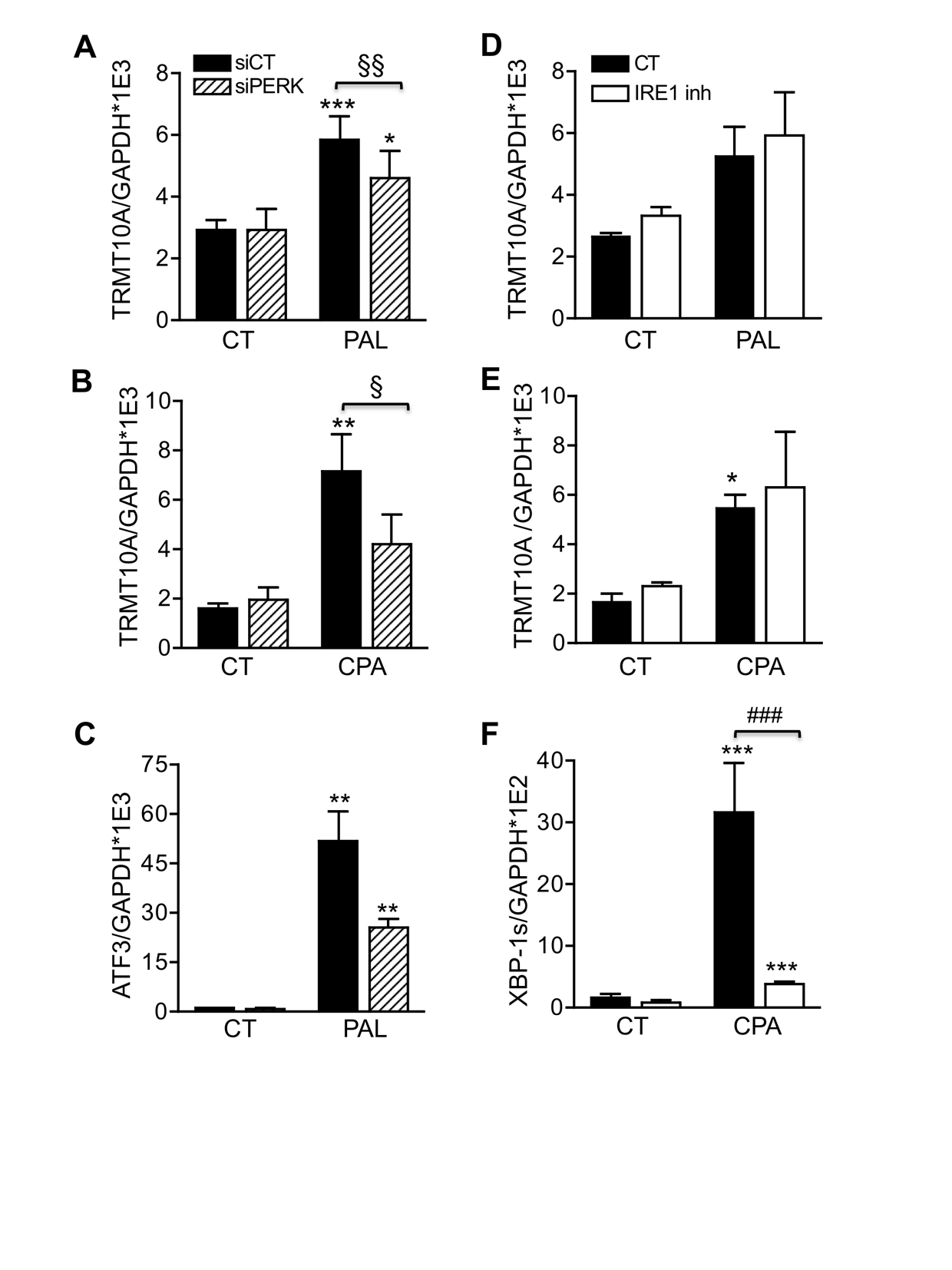

Supplement: Figure S9 — Endoplasmic reticulum stress modulates TRMT10A expression via PERK but not IRE1 activation. (A–C) INS-1E cells were transfected with control siRNA (siCT), or siRNA targeting rat PERK. 48 h after transfection cells were exposed or not to palmitate (PAL) or CPA for 16 h. (D–F) INS-1E cells were exposed for 16 h to palmitate (PAL) or CPA alone or combined with the IRE1 inhibitor 4μ8C. TRMT10A, ATF3 and XBP-1s mRNA expression was examined by real-time PCR and normalized to GAPDH expression (n = 4–6). * Treated vs CT, § siPERK vs siCT, ## IRE1 inhibitor vs CT. One symbol p<0.05, two p<0.01, three p<0.001, by ratio t test. (TIF) [file pgen.1003888.s009.tif]
