# Supplementary material for: tRNA Methyltransferase Homolog Gene TRMT10A Mutation in Young Onset Diabetes and Primary Microcephaly in Humans
Source: PLoS Genet. 2013 Oct 31;9(10):e1003888. doi: 10.1371/journal.pgen.1003888 (PMC3814312; doi:10.1371/journal.pgen.1003888)
Supplement: Table S1 — Sequence of rat and human siRNAs. (DOCX) [file pgen.1003888.s010.docx]

**Table S1**. Sequence of rat and human siRNAs

| **Species** | **Name** | **Source** | **Sequence (5’-3’)** |
| --- | --- | --- | --- |
| Rat | TRMT10A#1 | Invitrogen | CCUAUGUGAUUGGAGGGUUAGUGGA  UCCACUAACCCUCCAAUCACAUAGG |
| Rat | TRMT10A#2 | Invitrogen | CACGUUUAAGCAAGCUCCAGUUAU  AUAACUGGAUGCUUGCUUAAACGUG |
| Rat | PERK | Ambion | GUAUCCAUAUGACAACGGU |
| Human | TRMT10A#1 | Invitrogen | CAGAGCACUAUAGUGAACUCAUAAA  UUUAUGAGUUCACUAUAGUGCUCUG |
| Human | TRMT10A#2 | Invitrogen | GGUGAAGGGUGUGAACCAAUAUCUA  UAGAUAUUGGUUCACCCCUUCACC |
