# Supplementary material for: tRNA Methyltransferase Homolog Gene TRMT10A Mutation in Young Onset Diabetes and Primary Microcephaly in Humans
Source: PLoS Genet. 2013 Oct 31;9(10):e1003888. doi: 10.1371/journal.pgen.1003888 (PMC3814312; doi:10.1371/journal.pgen.1003888)
Supplement: Table S2 — Transfection reagents used in INS-1E cells, primary rat β-cells and human islets. (DOCX) [file pgen.1003888.s011.docx]

**Table S2**. Transfection reagents used in INS-1E cells, primary rat β-cells and human islets

| Cell type | Transfected sequence | Lipofectamine transfection agent | Plate | Transfection agent volume per well (μl) | Final volume (μl) |
| --- | --- | --- | --- | --- | --- |
| INS-1E | Vector | 2000 | 24 wells | 1.4 | 500 |
| INS-1E | Vector | 2000 | 8 well slides for microscopy | 0.7 | 250 |
| Dispersed rat islet cells | Vector | 2000 | 8 well slides | 0.87 | 250 |
| Dispersed human islets | Vector | 2000 | 8 well slides | 1.7 | 250 |
| INS-1E | siRNA | RNAiMAX | 24 wells | 1.0 | 500 |
| INS-1E | siRNA | RNAiMAX | 96 wells | 0.2 | 100 |
| Primary rat β-cells | siRNA | RNAiMAX | 96 wells | 0.25 | 100 |
| Dispersed human islets | siRNA | RNAiMAX | 96 wells | 0.4 | 100 |
