# Supplementary material for: tRNA Methyltransferase Homolog Gene TRMT10A Mutation in Young Onset Diabetes and Primary Microcephaly in Humans
Source: PLoS Genet. 2013 Oct 31;9(10):e1003888. doi: 10.1371/journal.pgen.1003888 (PMC3814312; doi:10.1371/journal.pgen.1003888)
Supplement: Table S3 — 5′-3′ primer sequence used for standard and real-time PCR. (DOCX) [file pgen.1003888.s012.docx]

**Table S3**. 5’-3’ primer sequence used for standard and real-time PCR

| **Species** | **Gene name** | **PRIMER forward** | **PRIMER reverse** |
| --- | --- | --- | --- |
| Rat-ST | TRMT10A | GATGAAACAGAGACTATGGGAAGA | TCGGACTTGATGTGAATATC |
| Rat-RT | TRMT10A | ATGACTTGATGGTGTTAAAG | ATCCATGTTCTTCTTCAGTTG |
| Rat-ST | GAPDH | ATGACTCTACCCACGGCAAG | TGTGAGGGAGATGCTCAGTG |
| Rat-RT | GAPDH | AGTTCAACGGCACAGTCAAG | TACTCAGCACCAGCATCACC |
| Rat-ST | OAZ1 | ATCCTCAACAGCCACTGCTT | CCAAGAAAGCTGAAGGTTCG |
| Rat-RT | OAZ1 | CACCATGCCGCTTCTTAGTC | CCGGACCCAGGTTACTACAG |
| Rat-ST | β-actin | atggtgggtatgggtcagaa | cagtgaggccaggatagagc |
| Rat-RT | β-actin | ctgtgcccatctatgagggt | CTCTCAGCTGTGGTGGTGAA |
| Rat-ST | CHOP | GTCTCTGCCTTTCGCCTTTG | CTACCCTCAGTCCCCTCCTC |
| Rat-RT | CHOP | CCAGCAGAGGTCACAAGCAC | CGCACTGACCACTCTGTTTC |
| Rat-ST | BiP | CTCAAAGAGCGCATTGACA | AATGCTATAGCCCAAGTGGCT |
| Rat-RT | BiP | CCACCAGGATGCAGACATTG | AGGGCCTCCACTTCCATAGA |
| Rat-ST | XBP-1s | AAACAGAGTAGCAGCGCAGACTGC | GGATCTCTAAGACTAGAGGCTTGGTG |
| Rat-RT | XBP-1s | GAGTCCGCAGCAGGTG | GCGTCAGAATCCATGGGA |
| Human-ST | β-actin | AAATCTGGCACCACACCTTC | CCGATCCACACGGAGTACTT |
| Human-RT | β-actin | CTGTACGCCAACACAGTGCT | GCTCAGGAGGAGCAATGATC |
| Human-ST | TRMT10A | AGAAGCCAAGATTAGGTGAAG | ACTAATCCTCCAATCACATAG |
| Human-RT | TRMT10A | ATGTTGTTCATAGCACCCTTC | ATGTTCTTTTTCAGCTGGCCT |
| Human-ST | ATF3 | ATCTCCTTCACCGTGGCTAC | AGGACCTGCCATCATACTGC |
| Human-RT | ATF3 | GCTGTCACCACGTGCAGTAT | TTTGTGTTAACGCTGGGAGA |
| Human-ST | BiP | GGATCTCTAAGACTAGAGGCTTGGTG | GGATCTCTAAGACTAGAGGCTTGGTG |
| Human-RT | BiP | QuantiTec HS HSPA5 (Qiagen) | QuantiTec HS HSPA5 (Qiagen) |
| Human-ST | XBP-1s | GGATCTCTAAGACTAGAGGCTTGGTG | GGATCTCTAAGACTAGAGGCTTGGTG |
| Human-RT | XBP-1s | CCGCAGCAGGTGCAGG | GAGTCAATACCGCAGAATCCA |

ST: standard, RT: real-time PCR
